# Colocalization analysis of polycystic ovary syndrome to identify potential disease-mediating genes and proteins

**DOI:** 10.1038/s41431-021-00835-8

**Published:** 2021-03-04

**Authors:** Jenny C. Censin, Jonas Bovijn, Michael V. Holmes, Cecilia M. Lindgren

**Affiliations:** 1 Big Data Institute at the Li Ka Shing Centre for Health Information and Discovery, University of Oxford, Oxford, UK; 2 Wellcome Centre for Human Genetics, University of Oxford, Oxford, UK; 3 NIHR Oxford Biomedical Research Centre, Oxford University Hospitals NHS Foundation Trust, John Radcliffe Hospital, Oxford, UK; 4 Medical Research Council Population Health Research Unit at the University of Oxford, Nuffield Department of Population Health, University of Oxford, Oxford, UK; 5 Clinical Trial Service Unit & Epidemiological Studies Unit (CTSU), Nuffield Department of Population Health, Big Data Institute Building, Roosevelt Drive, University of Oxford, Oxford, UK; 6 Program in Medical and Population Genetics, Broad Institute, Cambridge, MA USA; 7 Nuffield Department of Women’s and Reproductive Health, University of Oxford, Oxford, UK

**Keywords:** Endocrine reproductive disorders, Quantitative trait

## Abstract

Polycystic ovary syndrome (PCOS) is a common complex disease in women with a strong genetic component and downstream consequences for reproductive, metabolic and psychological health. There are currently 19 known PCOS risk loci, primarily identified in women of Han Chinese or European ancestry, and 14 of these risk loci were identified or replicated in a genome-wide association study of PCOS performed in up to 10,074 cases and 103,164 controls of European descent. However, for most of these loci the gene responsible for the association is unknown. We therefore use a Bayesian colocalization approach (Coloc) to highlight genes in PCOS-associated regions that may have a role in mediating the disease risk. We evaluated the posterior probabilities of evidence consistent with shared causal variants between 14 PCOS genetic risk loci and intermediate cellular phenotypes in one protein (*N* = 3301) and two expression quantitative trait locus datasets (*N* = 31,684 and *N* = 80–491). Through these analyses, we identified seven proteins or genes with evidence of a possibly shared causal variant for almost 30% of known PCOS signals, including follicle stimulating hormone and *ERBB3*, *IKZF4*, *RPS26*, *SUOX*, *ZFP36L2*, and *C8orf49*. Several of these potential effector proteins and genes have been implicated in the hypothalamic–pituitary–gonadal signalling pathway and provide an avenue for functional follow-up in order to demonstrate a causal role in PCOS pathophysiology.

## Introduction

Polycystic ovary syndrome (PCOS) is a common endocrinopathy, affecting between 6 and 10% of women of reproductive age [1], with consequences for reproductive, metabolic, and psychological health [2, 3]. There is evidence of a clear genetic component [4], and genome-wide association studies (GWASs) have identified 19 risk loci [5–9]. Some of these risk loci are close to genes with a plausible connection to PCOS pathophysiology, including genes involved in for example insulin and hypothalamic–pituitary–gonadal (HPG) signalling (e.g., *INSR*, the insulin receptor gene and *FSHR*, the FSH-receptor gene) [3, 6–8]. However, for most PCOS-associated loci the mediating genes and their functional effects remain to be identified and/or confirmed [6].

One approach to improve biological understanding of a disease risk locus is through colocalization analysis of the disease and intermediate cellular phenotypes, such as gene expression and protein levels in different tissues [10]. Colocalization analysis quantifies the probability that two traits share the same causal variant, and can thereby highlight genes and proteins that may mediate the risk of a disease [10]. We therefore investigated the evidence of colocalization between 14 PCOS-associated loci identified in a recent GWAS in Europeans [6] together with one study with protein and two studies with expression quantitative trait loci (pQTL and eQTL, respectively). Our results highlight several genes and proteins linked to the HPG axis and follicular development, including e.g. FSH, *ZFP36L2*, and *RAD50*, that may be of particular interest for further functional follow-up.

## Materials and methods

### Polycystic ovary syndrome dataset

**Fig. 1:**
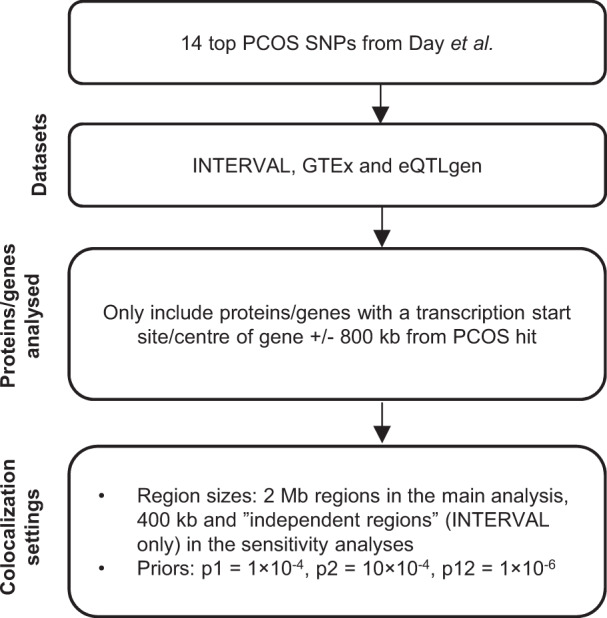
Study overview. Top SNPs associated with PCOS were extracted from Day et al. [6]. Colocalization was then performed between PCOS risk and both gene expression and protein levels, using data from INTERVAL [11, 12], GTEx [13], and eQTLgen [14]. Proteins and genes with a transcription start site or center of gene (depending on the gene/protein dataset) within 800 kb from a PCOS SNP were then analysed using the Bayesian colocalization software Coloc [10]. PCOS polycystic ovary syndrome; SNP single nucleotide polymorphism.

We obtained GWAS summary statistics for PCOS from Day et al. [6]. In their study, 14 genome-wide significant loci were identified in up to 10,074 cases and 103,164 controls of European ancestry (Fig. 1 and Table 1). Public summary statistics and single nucleotide polymorphism (SNP) estimates were available for (a) the 10,000 most robustly associated SNPs with estimates computed in the full sample and (b) for all SNPs with estimates computed in analyses excluding one of the cohorts (23andMe), resulting in a sample size of up to 4890 cases and 20,405 controls. We combined the two SNP summary statistics datasets to one dataset for use in the main analyses, with preference given to summary statistics computed using the full sample size. We then excluded SNPs found to be duplicated by position, missing relevant data, or indels.

**Table 1:** Summary statistics for the 14 PCOS SNPs.

SNP	Full SNP name	Chr	Pos	EA	NEA	EAF	Odds ratio (95% CI)	*P*
rs2178575:G>A	NC_000002.11:g.213391766G>A	2	213391766	A	G	0.15	1.18 (1.13–1.23)	3.34e−14
rs11031005:T>C	NC_000011.9:g.30226356T>C	11	30226356	C	T	0.15	1.17 (1.12–1.23)	8.66e−13
rs804279:A>T	NC_000008.10:g.11623889A>T	8	11623889	A	T	0.26	1.14 (1.10–1.18)	3.76e−12
rs11225154:G>A	NC_000011.9:g.102043240G>A	11	102043240	A	G	0.09	1.20 (1.13–1.26)	5.44e−11
rs9696009:G>A	NC_000009.11:g.126619233G>A	9	126619233	A	G	0.07	1.22 (1.15–1.30)	7.96e−11
rs13164856:T>C	NC_000005.9:g.131813204T>C	5	131813204	T	C	0.73	1.13 (1.09–1.18)	1.45e−10
rs1784692:T>C	NC_000011.9:g.113949232T>C	11	113949232	T	C	0.82	1.15 (1.10–1.21)	1.88e−10
rs7563201:G>A	NC_000002.11:g.43561780G>A	2	43561780	G	A	0.55	1.11 (1.08–1.15)	3.68e−10
rs8043701:A>T	NC_000016.9:g.52375777A>T	16	52375777	T	A	0.18	1.14 (1.09–1.18)	9.61e−10
rs1795379:T>C	NC_000012.11:g.75941042T>C	12	75941042	C	T	0.76	1.12 (1.08–1.17)	1.81e−09
rs853854:T>A	NC_000020.10:g.31420757T>A	20	31420757	T	A	0.50	1.10 (1.07–1.14)	2.36e−09
rs2271194:A>T	NC_000012.11:g.56477694A>T	12	56477694	A	T	0.42	1.10 (1.07–1.14)	4.57e−09
rs10739076:A>C	NC_000009.11:g.5440589A>C	9	5440589	A	C	0.31	1.12 (1.07–1.16)	2.51e−08
rs7864171:G>A	NC_000009.11:g.97723266G>A	9	97723266	G	A	0.57	1.10 (1.06-1.13)	2.95e−08

Summary statistics for the PCOS-associated SNPs that were identified or replicated in Day et al. [6]. *Chr* chromosome, *CI* confidence interval, *EA* effect allele, *NEA* non-effect allele, *Pos* position, *SNP* single nucleotide polymorphism.

### Protein and expression quantitative trait loci datasets

We used publicly available protein and expression genetic association data from the INTERVAL study [11, 12], the GTEx consortium [13], and the eQTLgen consortium [14]. pQTL data were taken from the INTERVAL study, which had performed GWASs for 2994 unique plasma proteins (3283 measured aptamers) in 3301 blood donors of European ancestry [11]. For GTEx, we used data from version 7, which contains *cis*-eQTL data for between 80 and 491 samples in 48 different tissues [13, 15]. Expression had been measured post-mortem, with ~85% of donors being of European (“White”) ancestry in the whole sample [15]. Lastly, the eQTLgen consortium had performed *cis*- and *trans*-eQTL analysis in up to 31,684 individuals, predominantly of European ancestry [14]. Both *cis*-associations, containing SNPs within 1 Mb from the centre of the gene, and trans-associations, containing SNPs over 5 Mb from the centre of the gene, are publicly available [14]. For all these datasets, we then excluded SNPs that were duplicated by position, missing relevant data, or indels.

### Colocalization analyses

We applied Coloc [10], a Bayesian test for colocalization, to evaluate the probability of evidence consistent with a shared causal signal between each PCOS risk loci and each p/eQTL (see Supplement). We performed colocalization using the coloc.abf() function in the Coloc R package, applying it to *cis*-genes using up to three different region sizes depending on QTL dataset.

For GTEx and eQTLgen, *cis*-association statistics were only available for SNPs within 1 Mb of the transcription start site and the centre of the gene, respectively [13, 14]. We therefore only analyzed genes and proteins with a transcription start site or centre of gene ± 800 kb of each top PCOS SNP (by *P* value) for all three QTL datasets, to ascertain that we had a sufficiently large region on both sides of the association peak to determine colocalization. We analyzed two different region sizes in GTEx and eQTLgen—the entire 2 Mb *cis*-region available in these datasets in the main analysis and a 400 kb region around the position of the top PCOS SNP as a sensitivity analysis. For GTEx, we only performed the analysis if the PCOS index SNP was present in the GTEx summary statistics for computational reasons. For colocalization analyses involving the protein data from the INTERVAL study [11], we evaluated three different region sizes—a 2 Mb region and a 400 kb region around the top SNP, as well as the top SNP’s “independent region” [16]. Independent regions were defined as the approximately independent regions of linkage disequilibrium in Europeans, as computed by Berisa et al. [17].

Coloc requires the assignment of prior probabilities for a SNP being associated with each trait (p1 and p2) and for a SNP being associated with both traits (p12). We set these prior probabilities to p1 = 1 × 10^−4^, p2 = 1 × 10^−4^, and p12 = 1 × 10^−6^, with the prior for p12 being more stringent than the default setting [10, 18].

Briefly, Coloc evaluates five different hypotheses. Hypothesis H_0_, H_1_ and H_2_ correspond to situations without associated/causal SNPs in both the PCOS and the protein/gene dataset, H_3_ to a situation where PCOS and the protein/gene have different associated/causal SNPs, and H_4_ where PCOS and the protein/gene have evidence consistent with a shared associated/causal SNP, i.e., colocalization [10]. Since we performed colocalization as a hypothesis-generating approach, all analyses with a colocalization posterior probability (PP) > 0.50 were seen as having nominal evidence of colocalization and analyzed further. A colocalization PP just above >0.50 should be regarded with caution, and we set the threshold for strong evidence of colocalization at PP ≥ 0.75. Power for detecting colocalization was computed as the sum of the PPs for hypothesis 3 (no colocalization) and hypothesis 4 (colocalization) [19].

### Additional analyses

We followed up colocalizing regions with assessing phenome-wide association study (PheWAS) data for the top PCOS SNP using the Open Target Genetics platform [20]. The significance threshold for a PheWAS association on the Open Targets Genetics platform is approximately *P* < 1 × 10^−5^ (based on visual inspection of the plotted threshold [20]). We further corrected for the six SNPs we investigated and set the threshold to *P* < 1.7 × 10^−6^ (1 × 10^−5^ corrected for six SNPs).

We also performed a range of sensitivity analyses. Analyses were reconducted using the PCOS dataset where the 23andMe cohort had been excluded, to have roughly the same sample size for all SNPs. We also computed the PP of colocalization using HyPrColoc [16], a recently developed extension of Coloc [10] (see Supplement). For these analyses we used the larger region sizes of 2 Mb for all three QTL datasets, as well as the independent regions for INTERVAL (according to HyPrColoc recommendations [16]). Finally, we applied an experimental method, “interaction-Coloc” (see Supplement for detailed rationale, methods and results). In brief, we first identified other genes/proteins linked to the genes/proteins identified in the main analysis, based on data from protein–protein interaction experiments. We then performed colocalization analysis for these “linked” genes/proteins with PCOS risk under the assumption that evidence of colocalization for two linked genes/proteins strengthens the evidence for a role of affiliated pathways in PCOS pathophysiology. For this analysis, we used the default Coloc priors given the links to the genes/proteins identified in the main analysis.

## Results

### Colocalization highlights genes with a potential mediating role

**Fig. 2:**
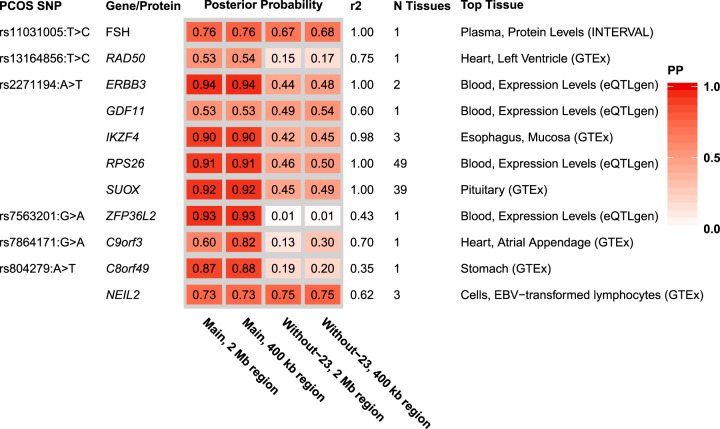
Posterior probabilities for genes and proteins with any evidence of colocalization. In the main approach, we used a region size spanning 2 Mb, and 400 kb regions as a sensitivity analysis. In addition to the main PCOS dataset, we also performed colocalization analysis with a PCOS dataset where the 23andMe cohort had been excluded as a sensitivity analysis. *r*^2^ is the linkage disequilibrium value between the top SNP in the main PCOS dataset and the top SNP in the tissue expression/protein dataset, using the 2 Mb region size. *N* tissues are the number of tissues where the colocalization PP > 0.5 in the main analysis. Only the results for the tissue with the highest posterior probability of colocalization in the main analysis are reported here (for full results and power calculations see Supplementary Tables 1–3). Gene-tissue combinations with a posterior probability of colocalization >0.50 were seen as having some evidence in favour of colocalization, whereas the threshold for strong evidence was set at ≥0.75. PCOS polycystic ovary syndrome, PP posterior probability, Without-23 the PCOS dataset where the 23andMe cohort had been excluded.

**Fig. 3:**
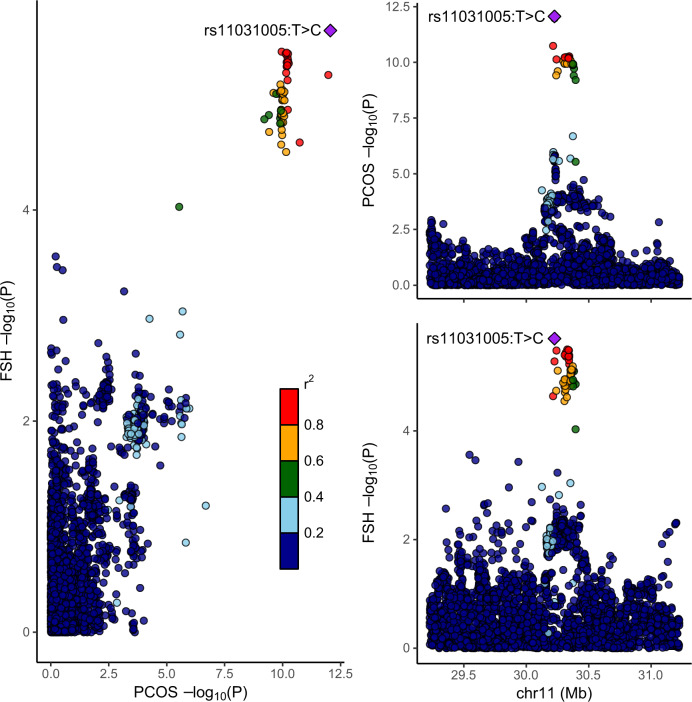
Associations between genetic variants and PCOS risk, using the main PCOS dataset and 2 Mb region sizes for FSH protein levels in blood. In each plot, each dot is a genetic variant. The SNP with the most significant *P* value for PCOS is marked, with the other SNPs colour-coded according to linkage disequilibrium (*r*^2^) in Europeans with the lead variant. SNPs with missing linkage disequilibrium information are also coded dark blue. In the left panel, −log10 *P* values for associations with PCOS risk are on the *x*-axes, and -log10 *P* values for associations with the protein levels on the *y*-axes. On the right panels, genomic positions are on the *x*-axes, and the *y*-axes show −log10 *P* values for PCOS on the upper panel and −log10 *P* values with the protein levels on the lower panel for the corresponding region. FSH follicle stimulating hormone; PCOS polycystic ovary syndrome; SNP single nucleotide polymorphism.

We identified seven proteins and genes with strong evidence of colocalization (PP ≥ 0.75), including the protein FSH, and the genes *SUOX*, *ERBB3*, *IKZF4*, *RPS26*, *C8orf49*, and *ZFP36L2* (Figs. 2–3 and Supplementary Tables 1–4; for a detailed description of genes see Supplement and Supplementary Figs. 1–10). In addition, four genes (*RAD50*, *GDF11*, *NEIL2*, and *C9orf3*) had nominal evidence of colocalization (PP > 0.50). Some of these genes and proteins, such as *RAD50*, had evidence of colocalization in only one tissue, whereas others, such as *RPS26* and *SUOX*, had evidence of colocalization in a large proportion of all tested tissues.

### Regulatory annotations and associations with other traits

The colocalization results had highlighted circulating FSH as colocalizing at the rs11031005:T>C locus (PP = 0.76) (see Fig. 3). We found that the rs11031005:T>C C-allele was associated with both higher PCOS risk (OR 1.17, 95% CI 1.12–1.23, P = 8.7 × 10^−13^) and lower FSH levels (−0.166 standard deviations, SE = 0.035, *P* = 2.0 × 10^−6^) [11]. The locus was also associated with several traits related to female hormonal regulation in the PheWAS look-up, with the two traits showing the most robust associations being length of menstrual cycle (*P* = 1.2 × 10^−42^) and age at menopause (*P* = 1.4 × 10^−15^) (Supplementary Table 5; for results for the other loci see Supplementary Tables 6–10) [20, 21].

Other PCOS loci were colocalized with the expression levels of several genes. At the rs2271194:A>T locus, the results supported colocalization for four genes (*ERBB3* PP = 0.94, *IKZF4* PP = 0.90, *RPS26* PP = 0.91, and *SUOX* PP = 0.92), as well as nominal evidence (PP = 0.53) for *GDF11* (Fig. 2, Supplementary Figs. 2–6). The PheWAS of this variant highlighted associations with a range of different traits, including e.g. obesity, household income, and haematologic traits (Supplementary Table 6) [20]. Look-up of rs2271194:A>T and its proxies (*r*^2^ > 0.8 in European-ancestry populations) in Haploreg [22] gave further evidence for a regulatory function in many different cell types, including e.g. immune cells, brain cells, and hepatocellular and cervical cancer cell lines.

### Sensitivity analyses

**Fig. 4:**
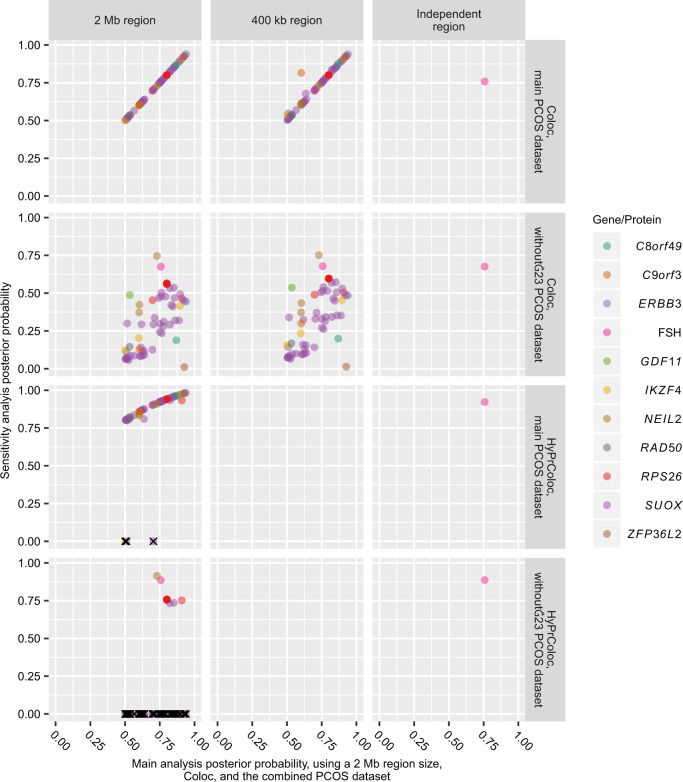
Plot showing the posterior probabilities as computed in the sensitivity analyses compared to those obtained in the main analysis. Top left plot is a reference plot with the main results on both axes. Multiple genes on each plot represent different tissues and/or datasets. Method-region-dataset combinations missing on plot were not performed due to either limitations in data availability or recommendations for HyPrColoc settings (see “Materials and methods”). HyPrColoc analyses that are not colocalizing do not have a posterior probability computed; these were manually assigned a posterior probability of 0 and marked with “X” on the plot. Without-23, PCOS dataset where the 23andMe cohort had been excluded.

We performed several sensitivity analyses. First, the number of SNPs included in an analysis can affect the PP of colocalization [18]. We therefore also conducted analyses using a region size of 400 kb for all three e/pQTL datasets [10, 18], as well as approximately independent regions of linkage disequilibrium [17] in INTERVAL (performed in INTERVAL only since the other datasets did not provide genome-wide summary statistics) [16]. This sensitivity analysis supported the main findings; all SNP-gene/protein-tissue combinations with evidence of colocalization (PP > 0.5) in the main analysis had a PP > 0.5 regardless of region size using the main PCOS dataset (Fig. 4).

Second, Coloc uses SNP-associations to compute PPs [10], and association statistics are dependent on sample size. We therefore performed colocalization analyses using estimates from the PCOS dataset where the 23andMe cohort had been excluded, to obtain a similar sample size for all SNPs. This analysis generally had lower power (possible range 0–1, with a power >0.80 indicating strong power) to detect colocalization, and generally a correspondingly lower PP of colocalization (Supplementary Tables 1–3 and Fig. 4) [19].

Next, we performed colocalization analysis using the software HyPrColoc [16]. Results using HyPrColoc also provided evidence for colocalization for all gene/protein-tissue combinations that were highlighted in the main analysis, except *ERBB3* in spinal cord, *IKZF4* in suprapubic skin, and *SUOX* in blood (but with evidence of colocalization between these genes and PCOS risk still present in other tissue types; Supplementary Tables 1, 2 and Fig. 4).

Finally, the experimental interaction-Coloc analyses provided suggestive evidence for two additional genes colocalizing with PCOS risk, highlighting their linked genes/proteins in the main analysis; FSH (as the linked gene coding for the FSH-receptor colocalized with PCOS risk at PP = 0.84) and *RAD50* (with its linked gene *UIMC1* colocalizing with PCOS risk at PP = 0.55) (see Supplement) [23].

## Discussion

Using a Bayesian colocalization approach, our results highlight several genes and proteins that may have a role in PCOS pathophysiology. We identify seven genes and proteins with strong evidence, and a further four genes and proteins with nominal evidence, of colocalization. Whereas potential mechanisms of action are unclear for some of the genes, half of the genes and proteins (FSH, *RAD50*, *ERBB3*, *RPS26*, and *ZFP36L2*) have links to the HPG axis and/or follicular development. We also find that a majority of the colocalizing genes are not the closest gene [6]. As the mediating genes for most of the genetic risk loci are still unclear [6], our results suggest genes with a higher likelihood of being involved in PCOS pathophysiology for functional follow-up.

The results highlighted FSH as a potential mediator at the rs11031005:T>C locus, corroborating the evidence of disruptions in gonadotropin signalling, specifically FSH and LH, contributing to PCOS pathophysiology [24]. FSH and LH are crucial hormones for follicular development and ovulation, and our PheWAS of the rs11031005:T>C locus showed an association with female reproductive traits [24]. LH and FSH share an alpha chain (encoded by *CGA* [25]), and disruption of *FSHB* has been associated with higher LH levels in humans [26]. It is thus possible that the PCOS association at the rs11031005:T>C locus may partly be caused by altered *FSHB* expression affecting LH levels. In addition, our results also implicated *ZFP36L2* at the rs7563201:G>A locus; another gene with links to gonadotropin signalling [27]. Female mice with a disruption in the *ZFP36L2* gene have disturbed oocyte maturation and ovulation, and its gene product has been implicated in regulation of LH-receptor levels [25, 27]. Previous studies have primarily suggested *THADA* to be the mediating gene at this locus [6, 28], but there was no evidence of *THADA* expression levels colocalizing with PCOS risk in any tissue in our study. We therefore suggest that *ZFP36L2* may be the mediating gene at the rs7563201:G>A locus and that the gene warrants further functional follow-up to evaluate a potential role in PCOS pathophysiology.

At the rs2271194:A>T locus, two of the colocalizing genes—*ERBB3* and *RPS26*—are likely candidates for mediating PCOS risk based on the literature, with both of them connected to the HPG axis. Gonadotropins have been shown to upregulate *ERBB3* expression and data suggest an important role in follicular development [29, 30]. The other gene, *RPS26*, has been implicated in DNA damage response and female fertility [25, 31, 32]. For example, oocyte-specific *Rps26*-knock-out mice have arrested oocyte growth, impaired follicle development, as well as poor response to gonadotropin stimulation [32]. Finally, we would like to highlight *RAD50* at the rs13164856:T>C locus. Female mice with disruptions in *RAD50* have reduced fertility [33] and the gene has been implicated in follicular development and oocyte development [34].

There are several strengths and limitations to our study. First, shared regulatory mechanisms between e.g. different genes and tissues can result in several gene/protein and tissue combinations colocalizing. However, it is unlikely that all of them are involved in disease development. The true mediating gene and tissue combination may not even have been investigated in the analyses, which may explain why some PCOS loci did not colocalize with any genes or proteins. In addition, we were surprised by the tissue types in which gene expression was colocalizing with PCOS. The disease is primarily thought of as being of hormonal and metabolic origin, wherefore we expected the results to highlight tissues types like the hypothalamus, the pituitary gland, and ovary and adipose tissue [2, 3]. Yet, many of the genes were colocalizing in seemingly unrelated tissue types, such as the heart (*RAD50* and *C9orf3)*. Still, for other genes, such as *RPS26*, even though the highest PP was achieved in the expression dataset with the highest sample size (whole blood in eQTLgen), the gene was also colocalizing with PCOS risk in ovary. Indeed, it is possible that the results in tissue types with many different cell types may have relatively lower PPs of colocalization if the disease is caused by changed expression in a single cell type in low abundance. Therefore, while colocalization can highlight genes, proteins, and tissues that are more likely to be involved in PCOS pathophysiology, results should be seen as hypothesis-generating rather than definitive evidence of a causal role. Second, if the causal SNP (or a proxy) is altering the coding sequence of a tested protein, it may become a false positive pQTL through changed aptamer binding. In our study, this could potentially result in rs11031005:T>C being a false pQTL for FSH, yet this is unlikely as the loci has previously been shown to associate with FSH levels using another protein quantification method [35]. Third, ancestral heterogeneity could potentially bias results due to different LD structure [10], even though all datasets primarily consisted of participants of European descent and we restricted our study to risk loci that were robust in a European-only GWAS [11, 13–15]. Fourth, the protein and expression datasets included both men and women [11, 13–15], whereas the PCOS GWAS [6] was performed in women only. If associations between genotypes and expression/protein levels differ between the sexes, it could bias results [36]. Fifth, Coloc also assumes a single causal variant per locus [10]. Accordingly, loci with multiple SNPs independently associated with either the disease or the intermediate trait may result in false negative colocalization results [10]. Still, Day et al. [6] did not report any multi-signal loci in the PCOS GWAS.

Finally, we performed a range of sensitivity analyses that largely supported the main results. We also presented an experimental method to nuance evidence, interaction-Coloc; however, we want to emphasize that the interaction-Coloc analyses should be interpreted with caution. The method has not been validated and is inherently limited by previously known interactions for each gene/protein, but we welcome future evaluations and developments of the method.

In summary, our results highlight potential mediating genes and proteins for almost a third of PCOS risk loci. Half of these genes and proteins have links to the HPG axis and follicular development, including the hormone FSH and the genes *ZFP36L2, ERBB3, RPS26*, and *RAD50*. In combination with previous studies that have indicated these genes as being involved in physiologic processes associated with PCOS, these genes may be of particular interest for further functional follow-up to assess if they have role in the disease development.

## Supplementary information

Supplement

Supplementary Tables

## Data Availability

Results are available in full in the supplementary tables and accessible at 10.6084/m9.figshare.13655444 (see Supplement for access to all other datasets used).
