# Supplementary material for: Colocalization analysis of polycystic ovary syndrome to identify potential disease-mediating genes and proteins
Source: Eur J Hum Genet. 2021 Mar 4;29(9):1446–54. doi: 10.1038/s41431-021-00835-8 (PMC8440598; doi:10.1038/s41431-021-00835-8)
Supplement: Supplementary file 1 — Supplement [file 41431_2021_835_MOESM1_ESM.docx]

**Supplementary Material**

Supplementary Material to “Colocalization analysis of polycystic ovary syndrome to identify potential disease-mediating genes and proteins” by Censin *et al.*

**Table of Contents**

[Detailed colocalization methods 2](#_Toc53660907)

[Evaluation of performing colocalization using the top 10,000 SNPs PCOS dataset 2](#_Toc53660908)

[Colocalization analyses using HyPrColoc 2](#_Toc53660909)

[Interaction-Coloc analyses 3](#_Toc53660910)

[Data availability 4](#_Toc53660911)

[Software 5](#_Toc53660912)

[Genes with evidence of colocalization only in sensitivity analyses 5](#_Toc53660913)

[Literature review of genes 5](#_Toc53660914)

[Supplementary Figures 8](#_Toc53660915)

[References 26](#_Toc53660916)

#

# Detailed colocalization methods

Gene positions and transcription start sites were determined using GRCh 37 and the biomaRt R package where needed (1,2). Minor allele frequencies from the polycystic ovary syndrome (PCOS) dataset were used in all Coloc analyses. For the number of cases and total sample size, we supplied 10,074 and 113,238 for the main PCOS dataset in the main analyses and 4,890 and 25,295 for the sensitivity-analysis excluding the 23andMe cohort (3). For INTERVAL and GTEx (4,5), we used the sample size reported for each tissue and dataset. For eQTLgen we supplied the average sample size for the included single-nucleotide polymorphisms (SNPs). As the eQTLgen summary statistics did not include effect estimates and standard errors, we let the coloc.abf() function approximate effect estimates from the P-values for this dataset (6,7). In addition, we computed linkage disequilibrium (LD) r^2^-values between the top SNPs in the main PCOS dataset and the expression/protein datasets using LDlinkR (CEU population in 1000 Genomes phase 3) (8,9).

# Evaluation of performing colocalization using the top 10,000 SNPs PCOS dataset

We first performed colocalization using only the 10,000 most robustly associated SNPs (based on analyses including the 23andMe cohort) dataset, in addition to using the main PCOS dataset and the dataset excluding the 23andMe cohort. We used the same settings as in the main colocalization analysis (see main article), and the results using the top 10,000 SNPs datasets were largely comparable to those using the main PCOS dataset. However, we realized that this approach could potentially yield false positive colocalization results.

One example of such a potential false positive is the colocalization analysis of *CAPS2* expression levels in transverse colon and PCOS risk (Supplementary Figure 11 and Supplementary Table 2). In the colocalization analysis using 2 Mb region sizes, there was nominal evidence in favour of colocalization using the top 10,000 SNPs PCOS dataset (posterior probability (PP)=0.56), but no evidence using the main dataset (PP=0).

Visual inspection of the region indicated that the light-blue SNPs (r^2^ between 0.2 and 0.4 with the top PCOS SNP) associated with *CAPS2* expression levels around -log10 P-values of 7.5-10 in Supplementary Figure 11B likely offer evidence against colocalization in the analysis using the main PCOS dataset. However, as these SNPs are not strongly associated with PCOS risk, they are excluded in the analysis using only the top 10,000 SNPs dataset (Supplementary Figure 11A).

We realized that if the two traits have two distinct causal variants in LD with each other, pre-selecting SNPs based on association with PCOS risk could result in the top expression quantitative trait loci (eQTLs) SNPs being excluded from the analysis. Thus, SNPs providing evidence against colocalization would not be included, which could yield false positive results. We have therefore not presented the colocalization results for the top 10,000 SNPs in the main section of the paper, but have included them in Supplementary Table 2 for full transparency.

# Colocalization analyses using HyPrColoc

For the HyPrColoc colocalization analyses, we used default priors (prior.1 = 1×10^-4^ and prior.2 = 0.98) but set both the regional and alignment probability thresholds to 0.8 (more stringent than default) (10).

As eQTLgen only provided Z-scores, we estimated betas and SEs using the formulas:

$$\hat{b} = z / \sqrt{2p(1-p)(n+z^{2})}$$

$$SE = 1 / \sqrt{2p(1-p)(n+z^{2})}$$

Where z is the Z-score, p is the minor allele frequency in the eQTLgen dataset and n is the sample size (6,11).

# Interaction-Coloc analyses

Several genes and proteins had evidence of colocalization in some loci (Fig 2), which could be due to shared regulatory mechanisms. In addition, identification of true causal genes/proteins is dependent on tissue- and timepoint relevant QTL datasets, an inherent problem in colocalization analyses (7,12). We therefore tested an exploratory approach, an “interaction-Coloc”-analysis, to further query the evidence for each colocalizing gene/protein and to nuance the evidence for each gene/protein.

In brief, we investigated if genes/proteins that are linked to the originally identified gene products and proteins provided additional evidence of their involvement in PCOS pathophysiology (see Supplementary Figure 12). Specifically, if there is evidence of colocalization with PCOS for two proteins (or their genes) known to interact with each other, we reasoned that this should increase the likelihood of them (or their genes) and their affiliated pathway mediating the relationship with the disease.

To identify proteins that interact with our primarily identified proteins or gene products, we downloaded data with protein-protein interactions in humans from Reactome (13) (available at <https://reactome.org/download/current/interactors/reactome.homo_sapiens.interactions.tab-delimited.txt>; for the exact level of evidence for each protein-protein interaction please see <https://reactome.org/>). Genes listed as part of proteins interacting with any of our proteins/gene products, and with ensembl gene identifiers, were extracted. For FSH, we only extracted interactions listed for the beta subunit (encoded by *FSHB*), since the alpha subunit (encoded by *CGA*) forms part of other hormones as well (14). We then extracted information of uniprot-identifiers, ensembl gene identifiers, gene positions and transcription start sites using GRCh 37 and the biomaRt R package to map between different datasets (1,2). We only included transcripts listed with a numeric autosomal chromosome and with information available in biomaRt. We included SNPs within +/- 1 Mb from the average transcription start site in the colocalization analyses using INTERVAL dataset (4), for the other datasets all available SNPs were used. We then applied Coloc (7), using the 2 Mb region sizes. As the genes and proteins in the interaction-Coloc analyses already had evidence of protein-protein interactions with the genes identified in the main analyses, we considered the prior probability of colocalization with PCOS higher and thus used a more lenient prior probability of colocalization than in the main analysis (p12 = 1×10^-5^, which is the same as the default setting in Coloc (7)).

We then performed colocalization for these “linked” genes/proteins (including both their genes and any gene products) with PCOS risk. Using this approach, we found evidence of colocalization for *FSHR* expression (interacting with FSH), and nominal evidence of colocalization for *UIMC1* (interacting with RAD50) and *RNF41* (interacting with ERBB3) (Supplementary Fig 1-2 and 13-14 and Supplementary Table 4) (13). However, as the genes *ERBB3* and *RNF41* are in the same locus this cannot be regarded as additional evidence for *ERBB3*.

This is a novel approach, but whereas it in theory should provide a more independent confirmation of a gene/protein being involved in the disease, the results should be interpreted with caution. Of the originally identified genes and proteins, only four (*C9orf3*, *ERBB3*, *FSH*, and *RAD50*) had interactors that we could perform colocalization on. As some had many known interactors and others none, this resulted in differing possibilities to identify colocalization for “linked” genes/proteins. In addition, even though the interaction-coloc analysis delivered plausible results and presents a possible extension of colocalization methodology, it has not been validated.

***Colocalization analysis of conditioned variants for* FSHR**

As the visual inspection of the *FSHR-*region (Supplementary Figure 14) indicated that there may be two or more independent eQTLs for *FSHR* expression in testis in the region, we performed colocalization analyses using estimates conditioned on top variants to determine which peak might be driving the colocalization.

***Linkage-disequilibrium reference panel for conditional analyses***

We constructed an LD-reference panel using a subset of the UK Biobank (15) and the imputed data. UK Biobank has a Research Tissue Bank approval (Research Ethics Committee reference 16/NW/0274, this study’s application ID 11867), and all participants gave informed consent. Briefly, we extracted a random subsample of 29,454 female participants in the “White British ancestry” subset, after excluding individuals that had withdrawn consent, mismatch between self-reported and genetically inferred sex, sex-chromosome aneuploidy, reported incompatible ancestries in different assessments, that were related to other individuals in the UK Biobank to a third degree or higher, heterozygosity or missingness outliers, or not included in the autosome phasing or in the kinship calculations (16). Genotype dosages were converted to best-guess genotypes using a hard-call threshold of 0.1. We excluded SNPs with imputation info score ≤0.3, minor allele frequency <0.01%, Hardy-Weinberg equilibrium exact test P<1×10^-6^, or genotype missing call rate >0.05. Analyses were done in plink versions 1.90b3 and 2.00a-20170724 (17).

***Conditional analyses***

We first performed clumping on the *FSHR-*locus for PCOS (options --clump-p1 0.0001, --clump-r2 0.05, and with --clump-kb spanning the entire locus), using the UK Biobank LD-reference panel. This identified two independent SNPs (rs2349415:T>C (NC_000002.11:g.49247832T>C) and rs4953650:C>T (NC_000002.11:g.49191739C>T), r^2^=0.04 in Europeans using 1000 Genomes (9,18)). We then computed estimates conditioned on each of these top SNPs using GCTA version 1.91.4 (option --cojo-cond) for both the *FSHR* eQTL dataset in testis and the main PCOS dataset (19,20). Information on effect allele frequencies for GTEx were taken from the file “GTEx_V7_cis_eqtl_summary.tar.gz (hg19)” (downloadable at http://cnsgenomics.com/software/smr/#DataResource) (11).

***Colocalization results for the conditional analyses***

The colocalization analyses indicated that the colocalization was driven by the rs4953650:C>T peak, as using estimates conditioned on rs2349415:T>C yielded a colocalization PP of 0.78, whereas there was no evidence of colocalization when conditioning on rs4953650:C>T (PP=0.02) (Supplementary Table 4, Supplementary Figure 15).

# Data availability

The PCOS genome-wide association study summary statistics are available at <https://www.repository.cam.ac.uk/handle/1810/283491> (3). The summary statistics from the INTERVAL study is available at <https://www.phpc.cam.ac.uk/ceu/proteins/> (4). Data from the eQTLgen consortia can be accessed at <https://molgenis26.gcc.rug.nl/downloads/eqtlgen/cis-eqtl> (6). The GTEx version 7 data are available at [https://gtexportal.org/](https://gtexportal.org/home/datasets) (5). Effect allele frequencies for GTEx were taken from the files “GTEx_V7_cis_eqtl_summary.tar.gz (hg19)” (downloadable at <http://cnsgenomics.com/software/smr/#DataResource>) (11). Independent regions as per Berisa *et al.* (21) can be accessed at <https://bitbucket.org/nygcresearch/ldetect-data/downloads/>. Genetic variants were matched to rsIDs as needed using the file “All_20180423.vcf.gz”, available at ftp://[ftp.ncbi.nih.gov/snp/organisms/human_9606_b151_GRCh37p13/VCF/](http://ftp.ncbi.nih.gov/snp/organisms/human_9606_b151_GRCh37p13/VCF/) (22). The PheWAS data were downloaded from the Open Targets Genetics website <https://genetics.opentargets.org> (23). In-silico functional investigations were done using Haploreg v4.1 at <https://pubs.broadinstitute.org/mammals/haploreg/haploreg.php> (24). Human protein-protein interactions from Reactome pathways is available at <https://reactome.org/download/current/interactors/reactome.homo_sapiens.interactions.tab-delimited.txt> (13). Individual-level data from UK Biobank cannot be shared publicly because of confidentiality but is available from the UK Biobank (<https://www.ukbiobank.ac.uk/>) for researchers who meet the criteria for access to confidential data. The UK Biobank has a Research Tissue Bank approval (Research Ethics Committee reference 16/NW/0274, this study’s application ID 11867) (15).

# Software

Analyses and plots were done using R versions 3.5.1 and 3.4.3 (25), bash version 4.1.2(2) (26), awk (27), and R packages coloc (7), hyprcoloc (10), LocusCompareR (28), tidyr (29), data.table (30), plyr (31), devtools (32), ggplot2 (33), and LDlinkR (8).

# Genes with evidence of colocalization only in sensitivity analyses

Four genes (*RCL1*, *TMEM123, ARL14EP*, and *ZC3H10*) had evidence of colocalization only when using other Coloc settings than the main approach, i.e. in the sensitivity analyses only (Supplementary Tables 1-2). We investigated these loci further, but the evidence of colocalization provided by visual inspection was in general weak and the genes had only weak support of a role in PCOS pathophysiology in the literature. For the interested reader, we have included a brief literature review of the genes below, together with those genes without detailed description in the main section of the paper.

# Literature review of genes

**rs13164856:T>C and *RAD50***

Another promising gene candidate is *RAD50*. The gene encodes DNA repair protein RAD50 (34), which together with MRE11 and another protein forms the MRE11 complex, which is involved in DNA damage response processes (35–40). Female mice with disruptions in the *Mre11* or *Rad50* genes have reduced fertility (36,40). It may be that the MRE complex affects oocyte elimination in the presence of DNA damage and thereby plays a part in follicular development and oocyte development (38). Even though our results only provided nominal evidence for involvement of *RAD50* in PCOS development, the evidence was strengthened by the interaction-Coloc analyses that also gave nominal colocalization evidence for another gene (*UIMC1*) implicated in the same DNA repair processes as the MRE11 complex (34,41) (see Supplementary Figure 1).

**rs2271194:A>T and *IKZF4***

*IKZF4* encodes the protein Zinc finger protein Eos (34), which plays a role in gene regulation in T-regulatory cells and the immune response (42). Deletions of Eos in T-regulatory cells in mice induces autoimmunity (43). Whereas the posterior probability for colocalization was high, there is little evidence in the literature to support a role for the gene in PCOS pathophysiology at present (Supplementary Tables 1-3, Supplementary Figure 4).

**rs2271194:A>T and *GDF11***

The protein Growth/differentiation factor 11 (GDF11) has been implicated in several mammalian developmental processes (44–46), including adipogenesis (47) and pancreatic β-cell development (48). The GDF11 protein has also been shown to improve β-cell function in cells and islets from mice (49). Both follicle stimulating hormone (FSH) and the GDF11 protein are regulated by follistatin (50–54), and there is evidence for follistatin levels being higher in PCOS patients (55). In addition, mice treated with the androgen dehydroepiandrosterone have down-regulated expression levels of *GDF11* in the ovary (56). It is possible that increased risk at the rs2271194:A>T locus is mediated through the effect of the GDF11 protein on adipogenesis and β-cell function and subsequent effects on e.g. insulin metabolism, or through yet unknown pathways in the ovary regulated by follistatin and/or androgen levels. However, more studies are needed to determine if GDF11 is involved in PCOS pathophysiology and, if so, the mechanistic pathways (see Supplementary Figure 3).

**rs2271194:A>T and *SUOX***

*SUOX* encodes mitochondrial sulfite oxidase (34), and it may be differentially expressed in oocytes from old versus young mice (57,58). The colocalization evidence for *SUOX* was comparable to the other genes in the rs2271194:A>T PCOS risk locus (Supplementary tables 1-2). Whereas visual inspection of the associations between PCOS risk and *SUOX* expression levels supported colocalization, it is unclear if and how *SUOX* might affect PCOS risk (Supplementary Figure 6).

**rs7864171:G>A and *C9orf3***

*C9orf3*, or *AOPEP* as the gene is also referred to, encodes Aminopeptidase O (34). Although the gene region has been linked to atrial fibrillation (59) and DNA methylation changes in twins born after in-vitro fertilization (60), the function of *C9orf3* is unclear. Our analyses indicated only moderate evidence of colocalization between *C9orf3* gene expression and PCOS risk (Supplementary Table 1-3 and Supplementary Figure 8).

**rs804279:A>T and *C8orf49***

*C8orf49* is a long non-coding RNA (34,61). Very little is known about the gene, although its expression levels were included in a recent expression signature for prognosis of endometrial cancer (61). The evidence for colocalization was relatively high using the main PCOS dataset regardless of region size, but only for expression levels in the stomach. The evidence of colocalization between *C8orf49* expression levels and PCOS risk in stomach diminished upon use of the PCOS dataset excluding the 23andMe cohort, in spite of high power, wherefore the results should be interpreted with caution (Supplementary tables 1-3, Supplementary Figure 9).

**rs804279:A>T and *NEIL2***

The gene *NEIL2* codes for the protein Endonuclease 8-like 2 (34). The protein is a base excision repair gene important for long-term genomic maintenance (62) and its expression is prognostic for resistance to endocrine therapy in estrogen receptor positive breast cancer (63). However, *Neil2* knock-out mice have normal fertility (62,64). Whereas DNA damage repair is crucial for normal oocyte development (65), one study indicated undetectable levels of *NEIL2* in human oocytes and blastocysts (66). However, *NEIL2* has also been implicated in DNA demethylation together with tet3 (67), and female mice with tet3-depletion have reduced fecundity and their oocytes a reduced ability to reprogram somatic cells (68). In summary, more studies are needed to determine if *NEIL2* has a role in PCOS pathophysiology (Supplementary tables 1-3, Supplementary Figure 10).

**rs10739076:A>C and *RCL1***

*RCL1* encodes the protein RNA 3'-terminal phosphate cyclase-like protein (34). The protein has been implicated in RNA processing (69), however not much else is known. In our colocalization analyses, there was only evidence for colocalization for the main PCOS dataset and blood expression levels in the +/- 200 kb region (Supplementary Table 1). Comparing the plots using the 2 Mb and 400 kb region sizes illustrates that part of the reason likely is a strong, second association peak for *RCL1* expression not present in the 400 kb region (Supplementary Figure 16). Still, visual inspection of the smaller region size reveals that there only seems to be a single shared SNP moderately associated with *RCL1* expression. Taken together, there is only weak evidence for colocalization between PCOS risk and *RCL1* gene expression.

**rs11031005:T>C and *ARL14EP***

The gene *ARL14EP* encodes the protein ARL14 effector protein (34). There is some evidence for the ARL14 effector protein being involved in MHC-class II molecule transportation (70), but it is unclear if it has any other function. There was evidence for *ARL14EP* colocalizing with PCOS risk, however primarily in testis and when using the PCOS dataset excluding the 23andMe cohort. The PP was less than 0.50 when using the main PCOS dataset regardless of region size. Given the available data, the evidence points to FSH rather than *ARL14EP* mediating the PCOS risk at the rs11031005:T>C PCOS risk locus.

**rs11225154:G>A and *TMEM123***

Porimin, also known as Transmembrane protein 123 and encoded by *TMEM123*, is a transmembrane protein that has been implicated in cell death (34). Whereas *TMEM123* seems to be expressed in cumulus cells in the oocytes of PCOS patients (71), there is otherwise little evidence to link it to PCOS at present. The colocalization evidence for *TMEM123* was weak, with the highest PP in any coloc analysis 0.54 in the brain cortex (Supplementary tables 1-2). In summary, there is little evidence to support a role of *TMEM123* in PCOS pathophysiology given our current knowledge.

**rs2271194:A>T and *ZC3H10***

Few studies have investigated the function of *ZC3H10* (encoding the protein Zinc finger CCCH domain-containing protein 10 (34)), but it has been suggested that the gene is an important regulator of mitochondrial energy metabolism (72). In addition, a loss-of-function mutation in humans has been associated with metabolic phenotypes, including higher body mass index and fasting glucose (72). In contrast to the other genes in the rs2271194:A>T PCOS risk locus, the evidence for colocalization between *ZC3H10* expression and PCOS risk was weak (Supplementary tables 1-2).

#

# Supplementary Figures


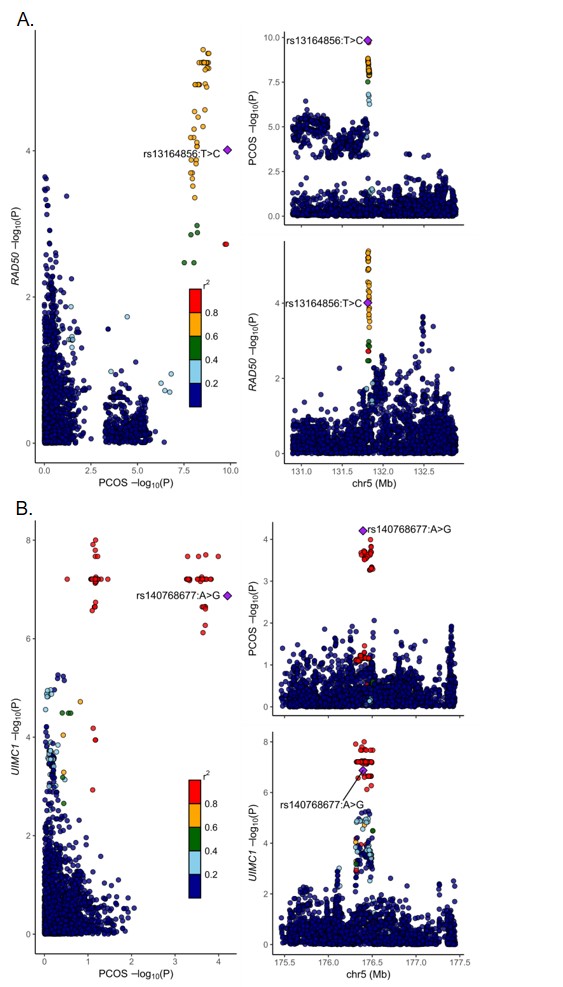


**Figure 1A and 1B. Associations between genetic variants and PCOS risk, using the main PCOS dataset and 2 Mb region sizes for (A) *RAD50* expression levels in left ventricle of the heart (B) *UIMC1* expression levels in blood (GTEx)**

In each plot, each dot is a genetic variant. The SNP with the most significant P-value for PCOS is marked, with the other SNPs colour-coded according to linkage disequilibrium (r^2^) in Europeans with the lead variant. SNPs with missing linkage disequilibrium information are also coded dark blue. In the left panels, -log10 P-values for associations with PCOS risk are on the x-axes, and -log10 P-values for associations with the expression levels on the y-axes. On the right panels, genomic positions are on the x-axes, and the y-axes show -log10 P-values for PCOS on the upper panel and -log10 P-values with the expression levels on the lower panel for the corresponding region. The full name for the rs140768677:A>G SNP is NC_000005.9:g.176395141A>G. PCOS, polycystic ovary syndrome; SNP, single nucleotide polymorphism.


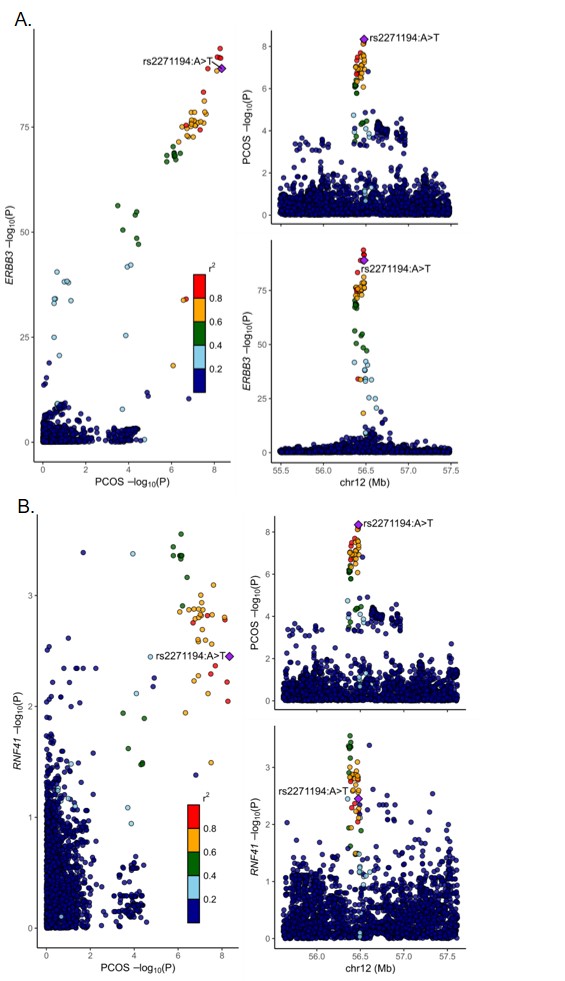


**Supplementary Figure 2A and 2B. Associations between genetic variants and PCOS risk, using the main PCOS dataset and 2 Mb region sizes for (A) *ERBB3* expression levels in blood (eQTLgen) (B) *RNF41* expression levels in coronary artery (GTEx)**

In each plot, each dot is a genetic variant. The SNP with the most significant P-value for PCOS is marked, with the other SNPs colour-coded according to linkage disequilibrium (r^2^) in Europeans with the lead variant. SNPs with missing linkage disequilibrium information are also coded dark blue. In the left panels, -log10 P-values for associations with PCOS risk are on the x-axes, and -log10 P-values for associations with the expression levels on the y-axes. On the right panels, genomic positions are on the x-axes, and the y-axes show -log10 P-values for PCOS on the upper panel and -log10 P-values with the expression levels on the lower panel for the corresponding region. PCOS, polycystic ovary syndrome; SNP, single nucleotide polymorphism.


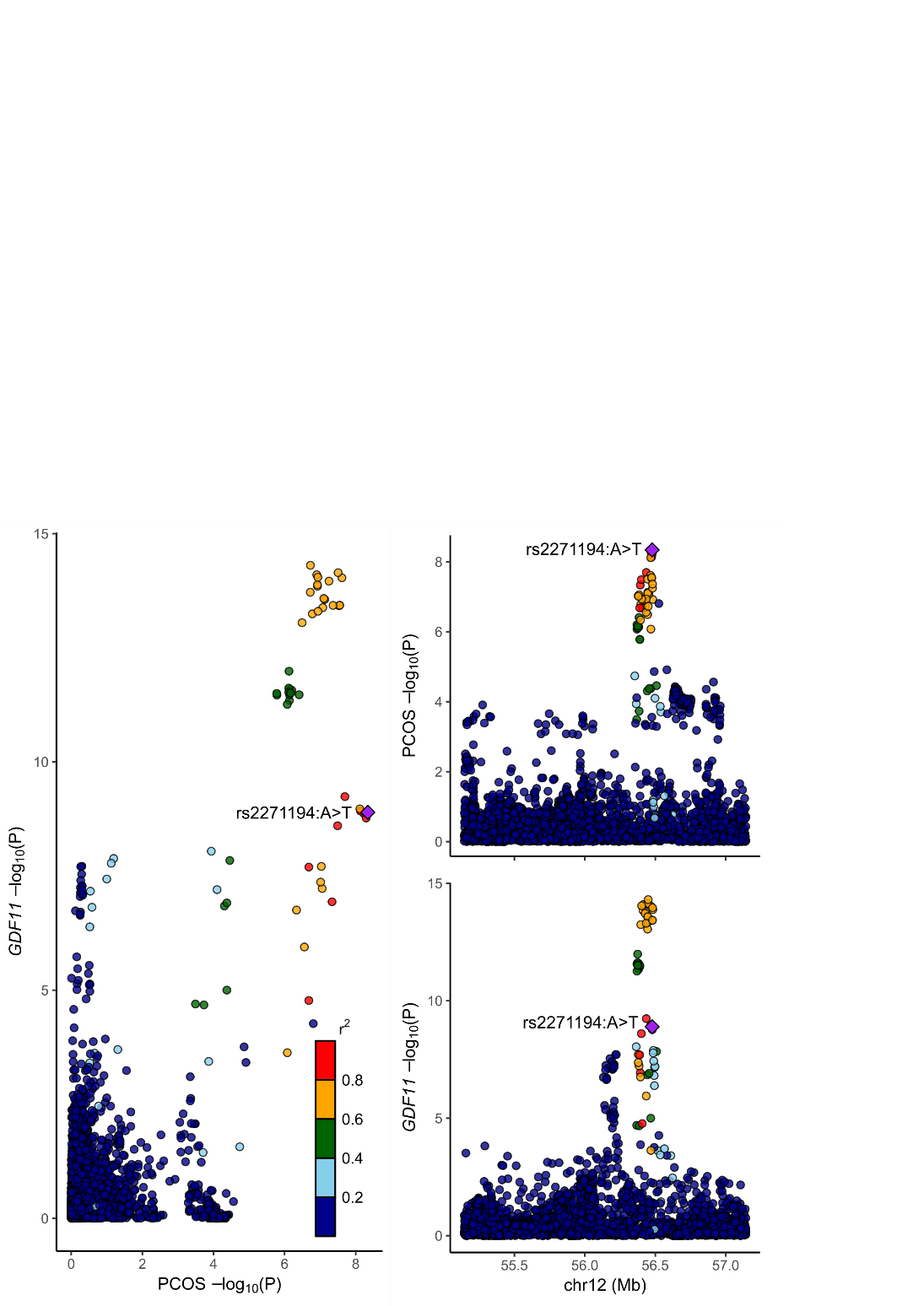


**Supplementary Figure 3. Associations between genetic variants and PCOS risk, using the main PCOS dataset and 2 Mb region sizes for *GDF11* expression levels in blood (eQTLgen)**

In each plot, each dot is a genetic variant. The SNP with the most significant P-value for PCOS is marked, with the other SNPs colour-coded according to linkage disequilibrium (r^2^) in Europeans with the lead variant. SNPs with missing linkage disequilibrium information are also coded dark blue. In the left panel, -log10 P-values for associations with PCOS risk are on the x-axis, and -log10 P-values for associations with the expression levels on the y-axes. On the right panels, genomic positions are on the x-axes, and the y-axes show -log10 P-values for PCOS on the upper panel and -log10 P-values with the expression levels on the lower panel for the corresponding region. PCOS, polycystic ovary syndrome; SNP, single nucleotide polymorphism.


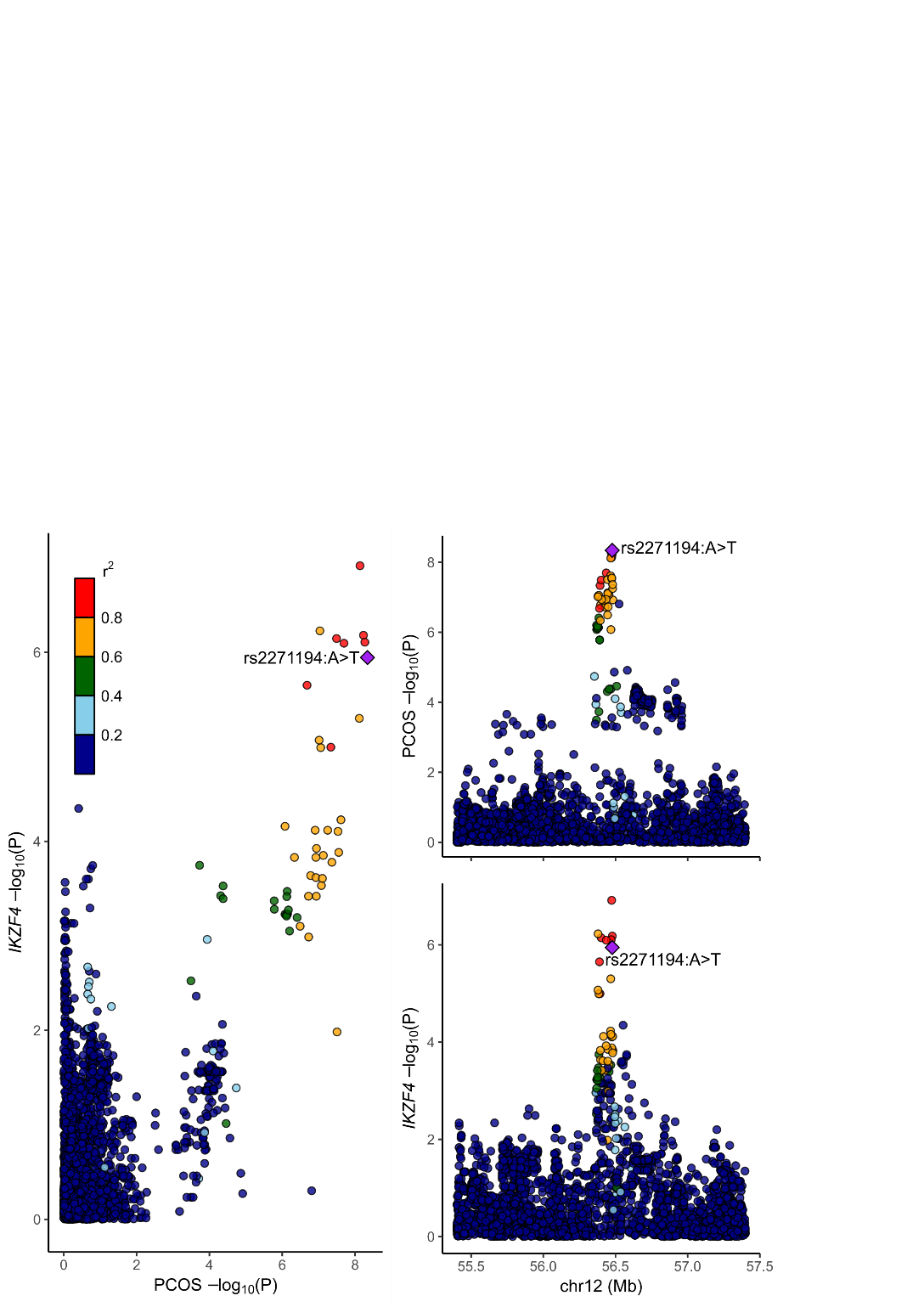


**Supplementary Figure 4. Associations between genetic variants and PCOS risk, using the main PCOS dataset and 2 Mb region sizes for *IKZF4* expression levels in esophagus (mucosa)**

In each plot, each dot is a genetic variant. The SNP with the most significant P-value for PCOS is marked, with the other SNPs colour-coded according to linkage disequilibrium (r^2^) in Europeans with the lead variant. SNPs with missing linkage disequilibrium information are also coded dark blue. In the left panel, -log10 P-values for associations with PCOS risk are on the x-axis, and -log10 P-values for associations with the expression levels on the y-axes. On the right panels, genomic positions are on the x-axes, and the y-axes show -log10 P-values for PCOS on the upper panel and -log10 P-values with the expression levels on the lower panel for the corresponding region. PCOS, polycystic ovary syndrome; SNP, single nucleotide polymorphism.


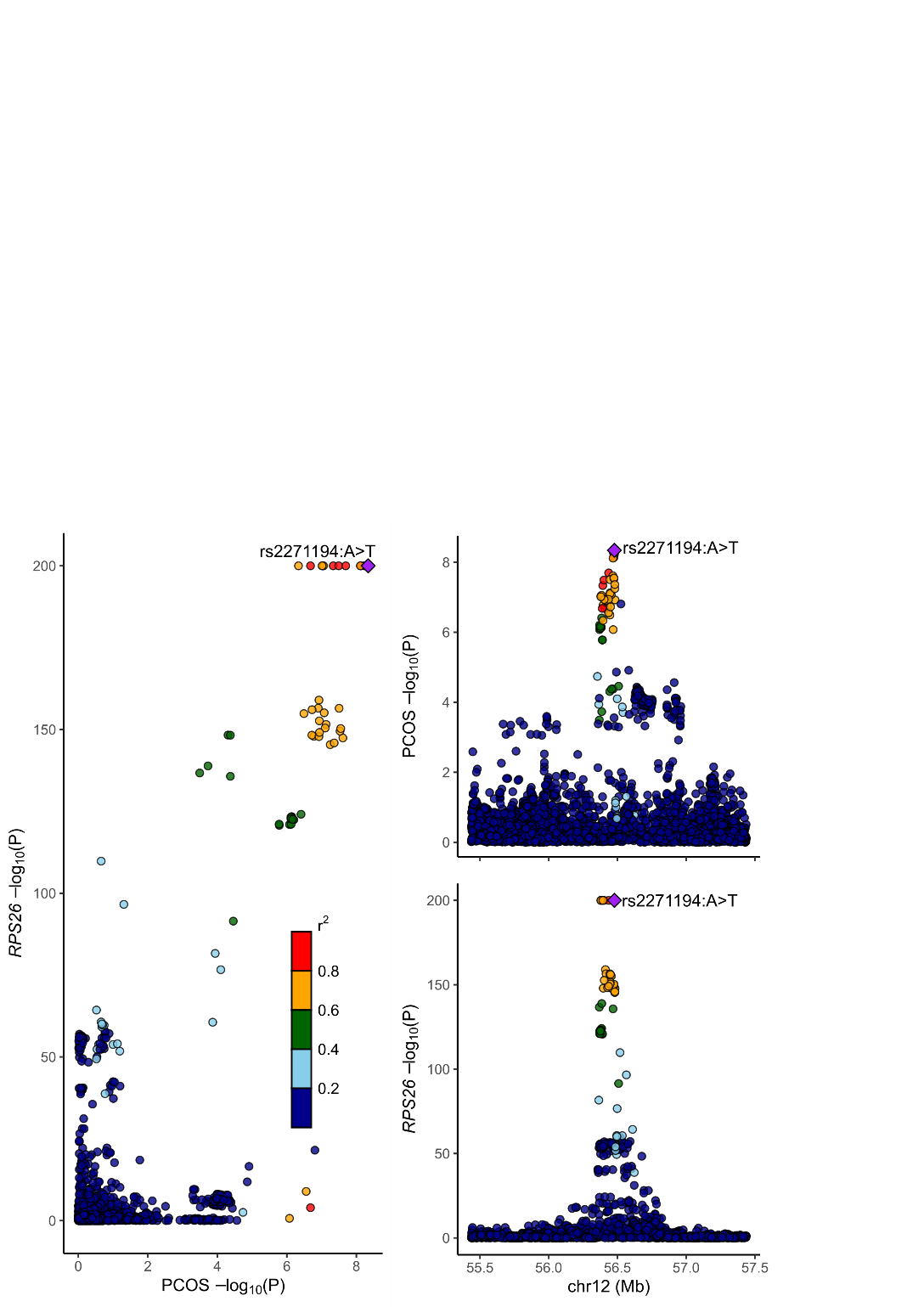


**Supplementary Figure 5. Associations between genetic variants and PCOS risk, using the main PCOS dataset and 2 Mb region sizes for *RPS26* expression levels in blood (eQTLgen)**

In each plot, each dot is a genetic variant. The SNP with the most significant P-value for PCOS is marked, with the other SNPs colour-coded according to linkage disequilibrium (r^2^) in Europeans with the lead variant. SNPs with missing linkage disequilibrium information are also coded dark blue. In the left panel, -log10 P-values for associations with PCOS risk are on the x-axis, and -log10 P-values for associations with the expression levels on the y-axes. We manually assigned P-values <1×100^-200^ to 1×100^-200^ for plotting. On the right panels, genomic positions are on the x-axes, and the y-axes show -log10 P-values for PCOS on the upper panel and -log10 P-values with the expression levels on the lower panel for the corresponding region. PCOS, polycystic ovary syndrome; SNP, single nucleotide polymorphism.


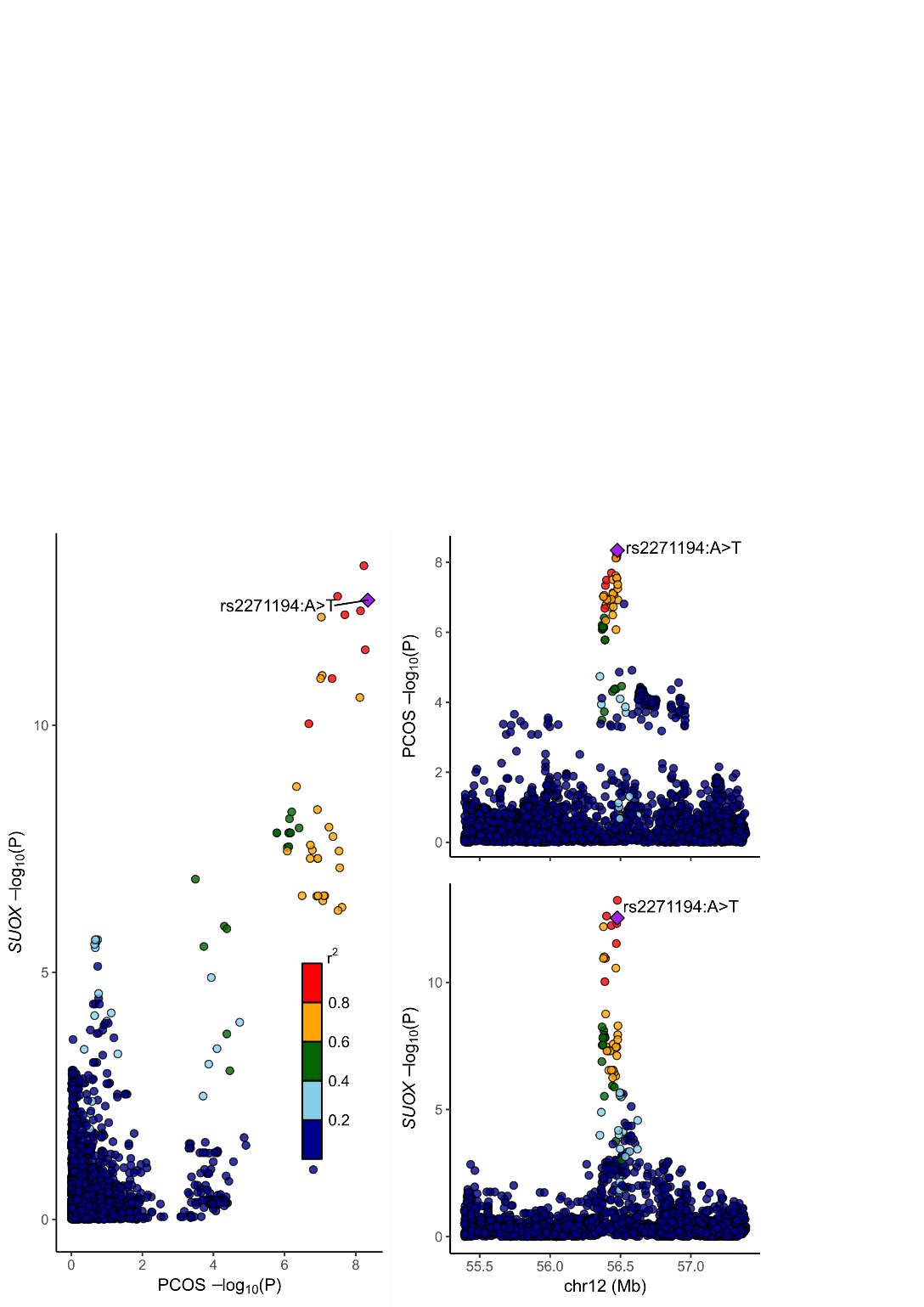


**Supplementary Figure 6. Associations between genetic variants and PCOS risk, using the main PCOS dataset and 2 Mb region sizes for *SUOX* expression levels in pituitary**

In each plot, each dot is a genetic variant. The SNP with the most significant P-value for PCOS is marked, with the other SNPs colour-coded according to linkage disequilibrium (r^2^) in Europeans with the lead variant. SNPs with missing linkage disequilibrium information are also coded dark blue. In the left panel, -log10 P-values for associations with PCOS risk are on the x-axis, and -log10 P-values for associations with the expression levels on the y-axes. On the right panels, genomic positions are on the x-axes, and the y-axes show -log10 P-values for PCOS on the upper panel and -log10 P-values with the expression levels on the lower panel for the corresponding region. PCOS, polycystic ovary syndrome; SNP, single nucleotide polymorphism.


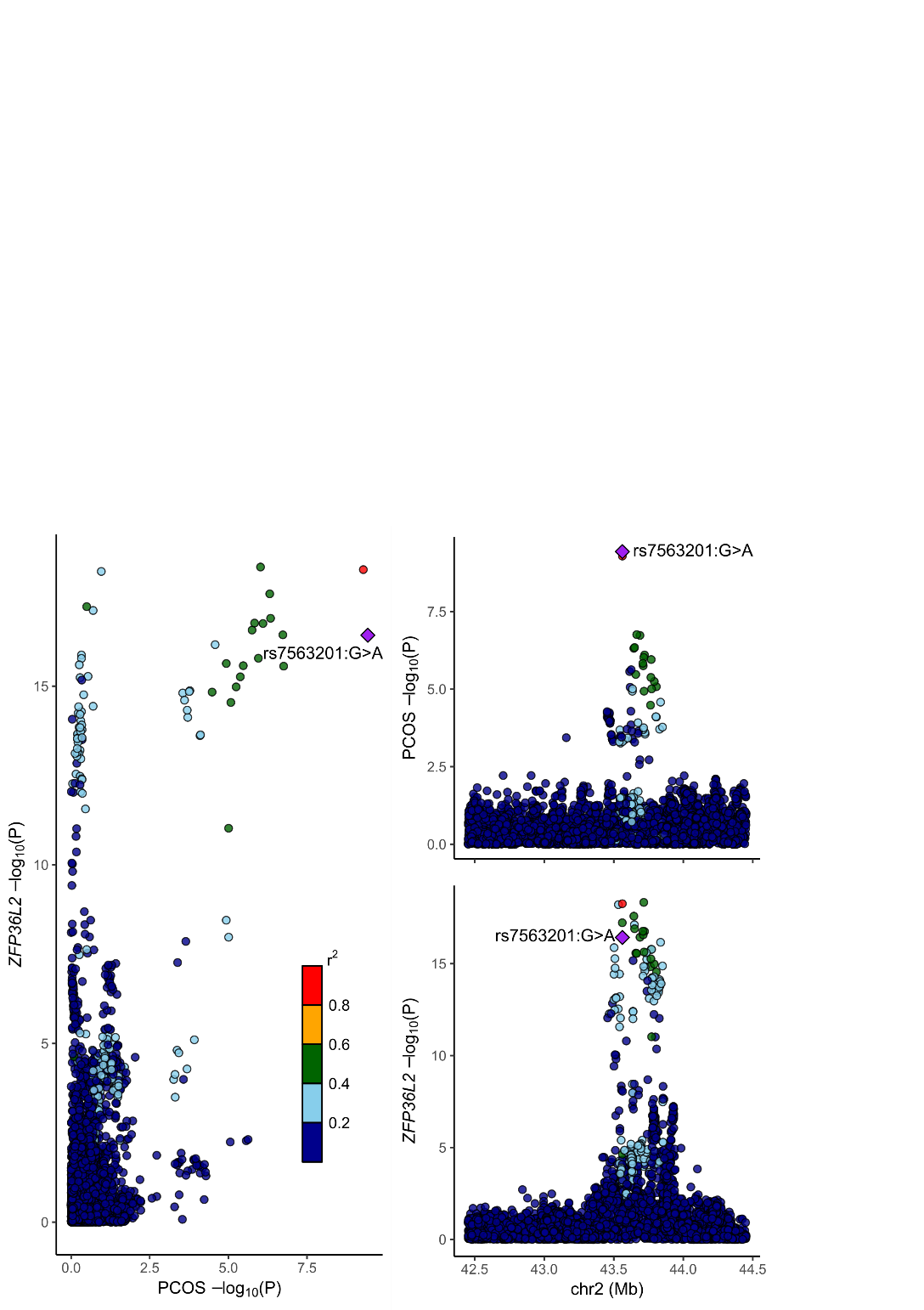


**Supplementary Figure 7. Associations between genetic variants and PCOS risk, using the main PCOS dataset and 2 Mb region sizes for *ZFP36L2* expression levels in blood (eQTLgen)**

In each plot, each dot is a genetic variant. The SNP with the most significant P-value for PCOS is marked, with the other SNPs colour-coded according to linkage disequilibrium (r^2^) in Europeans with the lead variant. SNPs with missing linkage disequilibrium information are also coded dark blue. In the left panel, -log10 P-values for associations with PCOS risk are on the x-axis, and -log10 P-values for associations with the expression levels on the y-axes. On the right panels, genomic positions are on the x-axes, and the y-axes show -log10 P-values for PCOS on the upper panel and -log10 P-values with the expression levels on the lower panel for the corresponding region. PCOS, polycystic ovary syndrome; SNP, single nucleotide polymorphism.


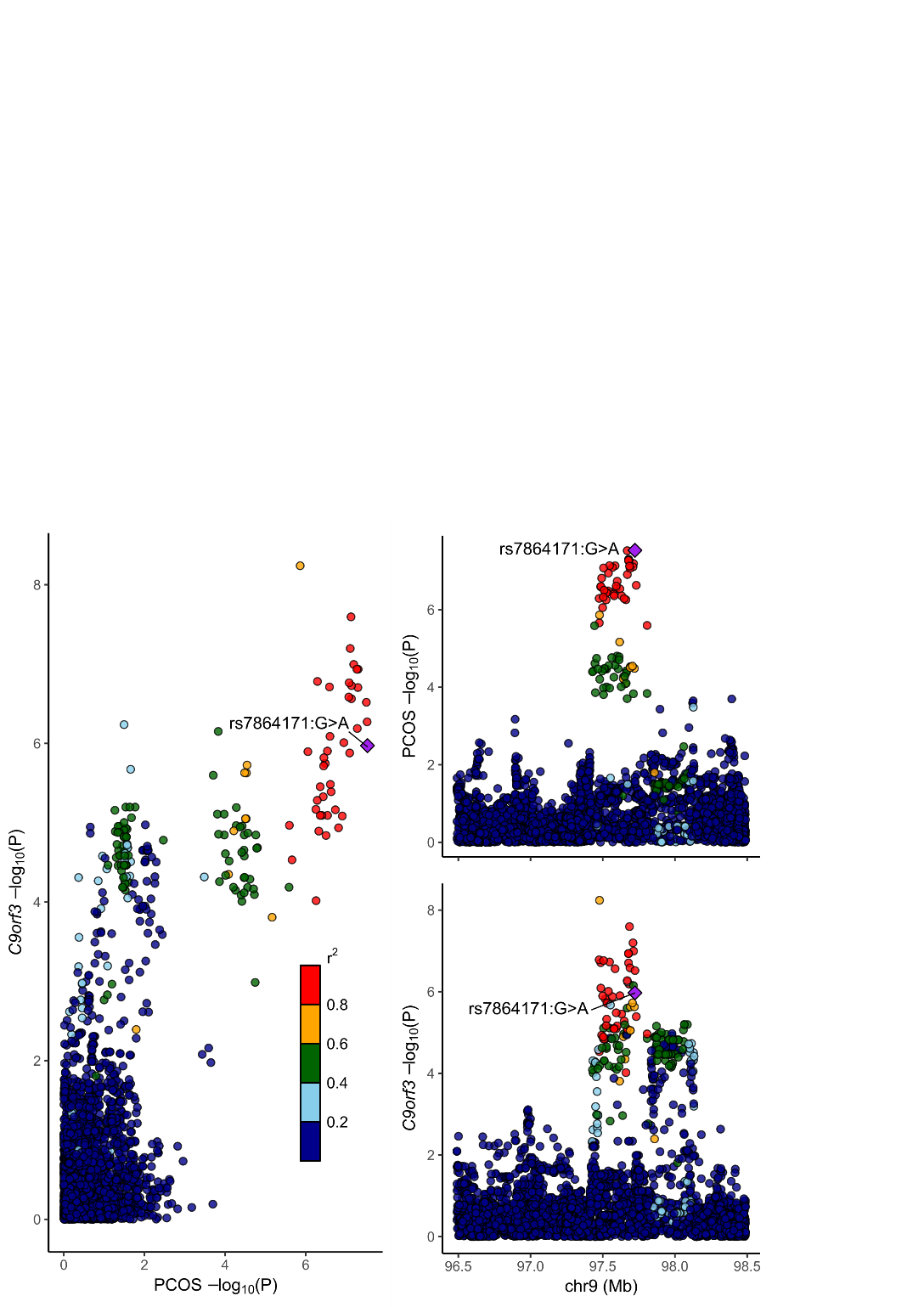


**Supplementary Figure 8. Associations between genetic variants and PCOS risk, using the main PCOS dataset and 2 Mb region sizes for *C9orf3* expression levels in atrial appendage of the heart**

In each plot, each dot is a genetic variant. The SNP with the most significant P-value for PCOS is marked, with the other SNPs colour-coded according to linkage disequilibrium (r^2^) in Europeans with the lead variant. SNPs with missing linkage disequilibrium information are also coded dark blue. In the left panel, -log10 P-values for associations with PCOS risk are on the x-axis, and -log10 P-values for associations with the expression levels on the y-axes. On the right panels, genomic positions are on the x-axes, and the y-axes show -log10 P-values for PCOS on the upper panel and -log10 P-values with the expression levels on the lower panel for the corresponding region. PCOS, polycystic ovary syndrome; SNP, single nucleotide polymorphism.


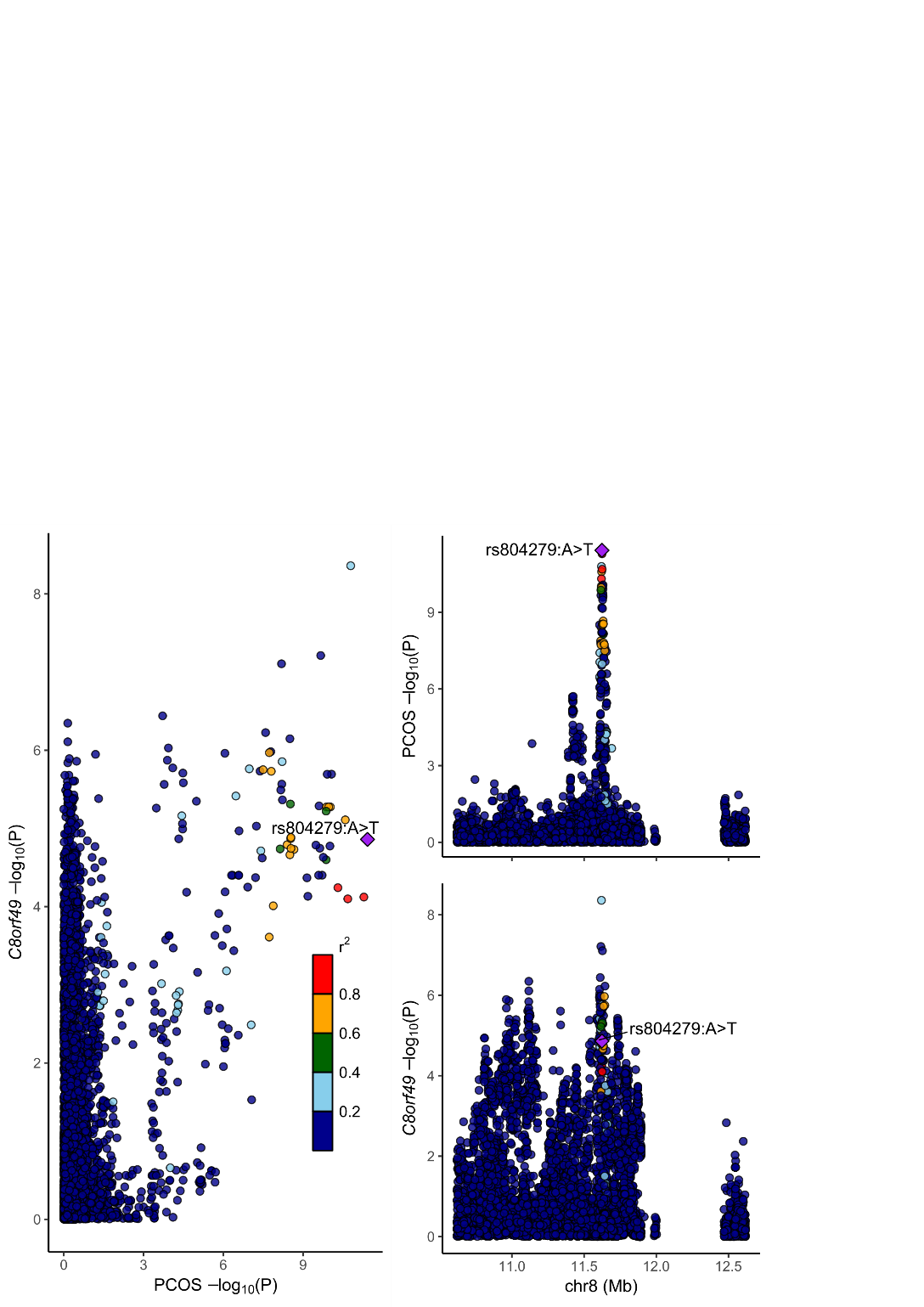


**Supplementary Figure 9. Associations between genetic variants and PCOS risk, using the main PCOS dataset and 2 Mb region sizes for *C8orf49* expression levels in stomach**

In each plot, each dot is a genetic variant. The SNP with the most significant P-value for PCOS is marked, with the other SNPs colour-coded according to linkage disequilibrium (r^2^) in Europeans with the lead variant. SNPs with missing linkage disequilibrium information are also coded dark blue. In the left panel, -log10 P-values for associations with PCOS risk are on the x-axis, and -log10 P-values for associations with the expression levels on the y-axes. On the right panels, genomic positions are on the x-axes, and the y-axes show -log10 P-values for PCOS on the upper panel and -log10 P-values with the expression levels on the lower panel for the corresponding region. The region included an area without SNPs, spanning around 12-12.5 Mb on chromosome 8. A look-up using the UCSC genome browser (73) showed that the region had very poor mappability, explaining the lack of data. PCOS, polycystic ovary syndrome; SNP, single nucleotide polymorphism.


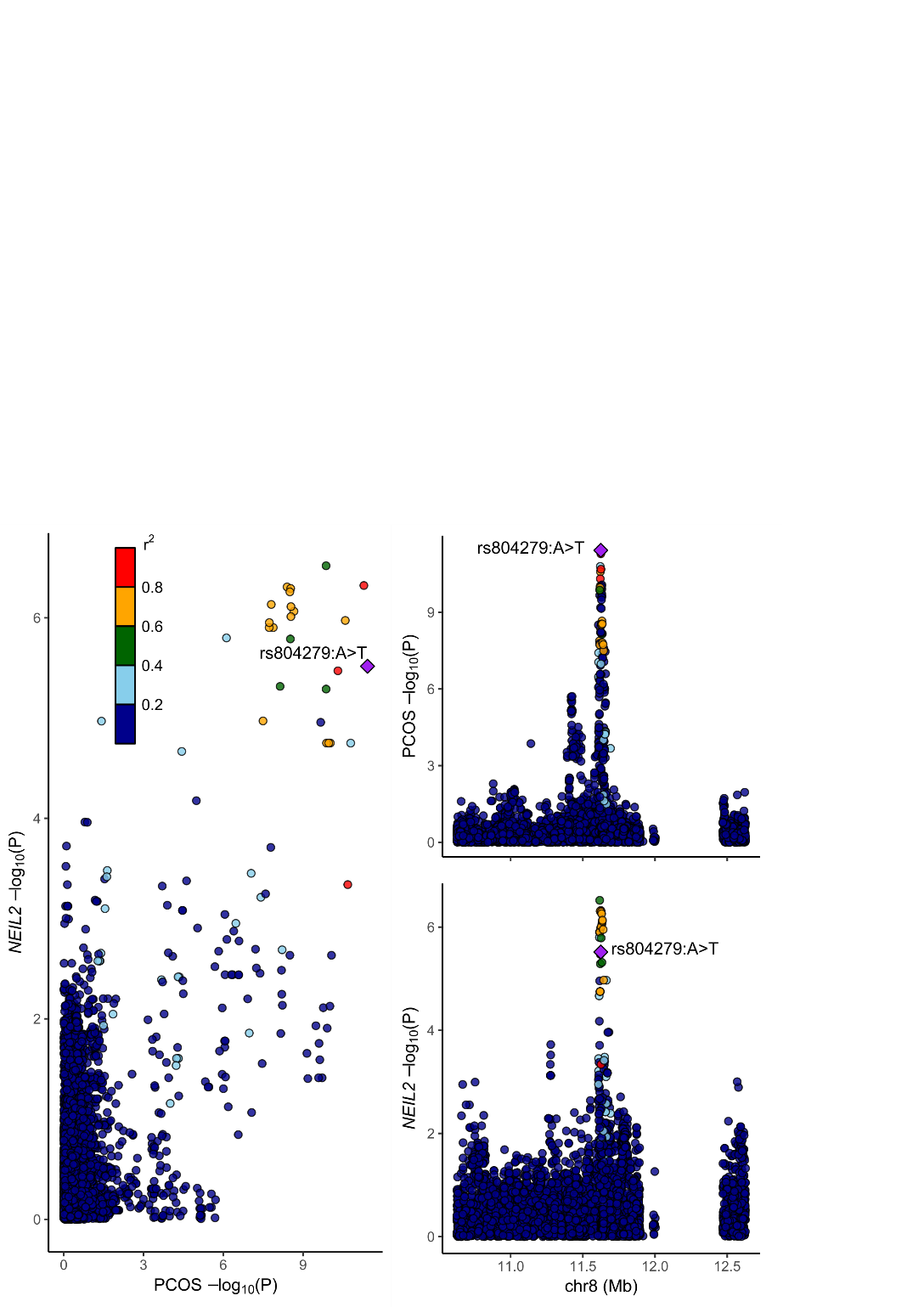


**Supplementary Figure 10. Associations between genetic variants and PCOS risk, using the main PCOS dataset and 2 Mb region sizes for *NEIL2* expression levels in EBV-transformed lymphocytes**

In each plot, each dot is a genetic variant. The SNP with the most significant P-value for PCOS is marked, with the other SNPs colour-coded according to linkage disequilibrium (r^2^) in Europeans with the lead variant. SNPs with missing linkage disequilibrium information are also coded dark blue. In the left panel, -log10 P-values for associations with PCOS risk are on the x-axis, and -log10 P-values for associations with the expression levels on the y-axes. On the right panels, genomic positions are on the x-axes, and the y-axes show -log10 P-values for PCOS on the upper panel and -log10 P-values with the expression levels on the lower panel for the corresponding region. The region included an area without SNPs, spanning around 12-12.5 Mb on chromosome 8. A look-up using the UCSC genome browser (73) showed that the region had very poor mappability, explaining the lack of data. PCOS, polycystic ovary syndrome; SNP, single nucleotide polymorphism.


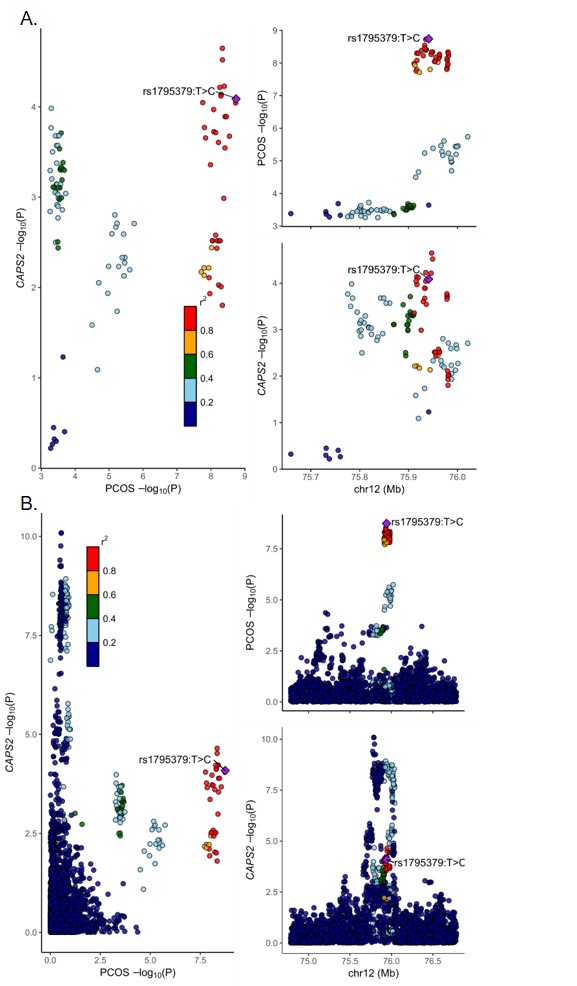


**Supplementary Figure 11A and 11B. Associations between genetic variants and PCOS risk for *CAPS2* expression levels in transverse colon using (A) the top 10,000 SNPs PCOS dataset (B) the main PCOS dataset**

In each plot, each dot is a genetic variant. The SNP with the most significant P-value for PCOS is marked, with the other SNPs colour-coded according to linkage disequilibrium (r^2^) in Europeans with the lead variant. SNPs with missing linkage disequilibrium information are also coded dark blue. In the left panels, -log10 P-values for associations with PCOS risk are on the x-axes, and -log10 P-values for associations with the protein/transcript levels on the y-axes. On the right panels, genomic positions are on the x-axes, and the y-axes show -log10 P-values for PCOS on the upper panel and -log10 P-values with the protein/expression levels on the lower panel for the corresponding region. PCOS, polycystic ovary syndrome; SNP, single nucleotide polymorphism.


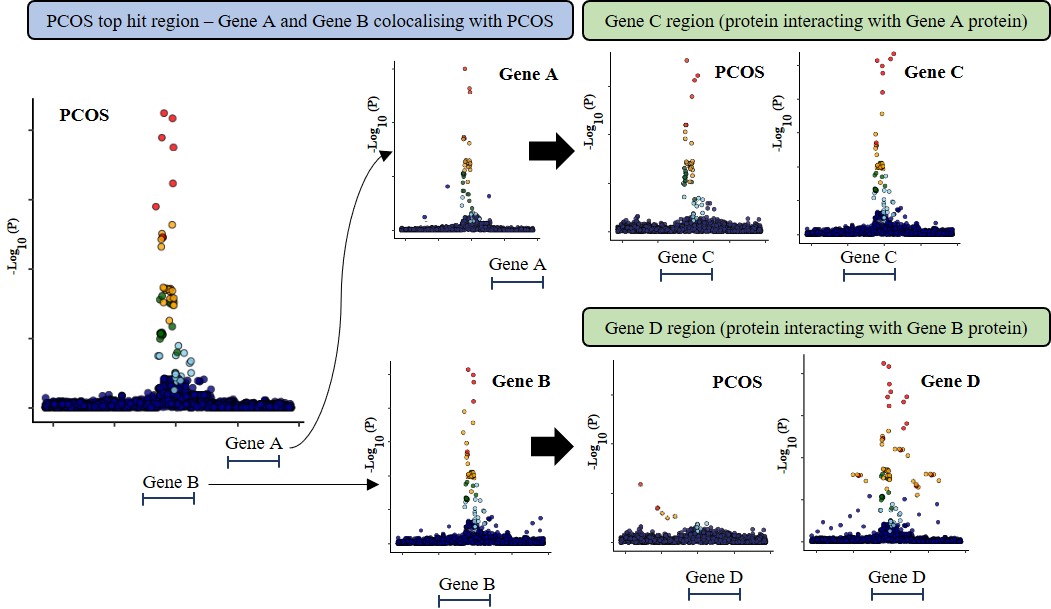


**Supplementary Figure 12. A hypothetical example explaining the rationale for investigating the evidence for colocalization between PCOS and the protein/expression levels of other genes linked to the originally identified genes/proteins.**

For a region associated with PCOS P<5×10^-8^, quantitative trait loci (QTLs) of Gene A and Gene B are both colocalizing with PCOS risk, possibly due to shared regulatory mechanisms. To investigate the evidence for each gene, the evidence for colocalization between PCOS and gene expression/protein levels for proteins with evidence of interaction with proteins of Gene A (denoted Gene C) and Gene B (denoted Gene D) are evaluated. If the QTL for Gene C colocalizes with PCOS (even though the top PCOS signal in the region of Gene C has a P-value >5×10^-8^), whereas there is no colocalization between the QTL for Gene D and PCOS, we reasoned that this would provide more evidence for Gene A rather than Gene B being implicated in PCOS pathophysiology. PCOS, polycystic ovary syndrome; QTL, quantitative trait locus.


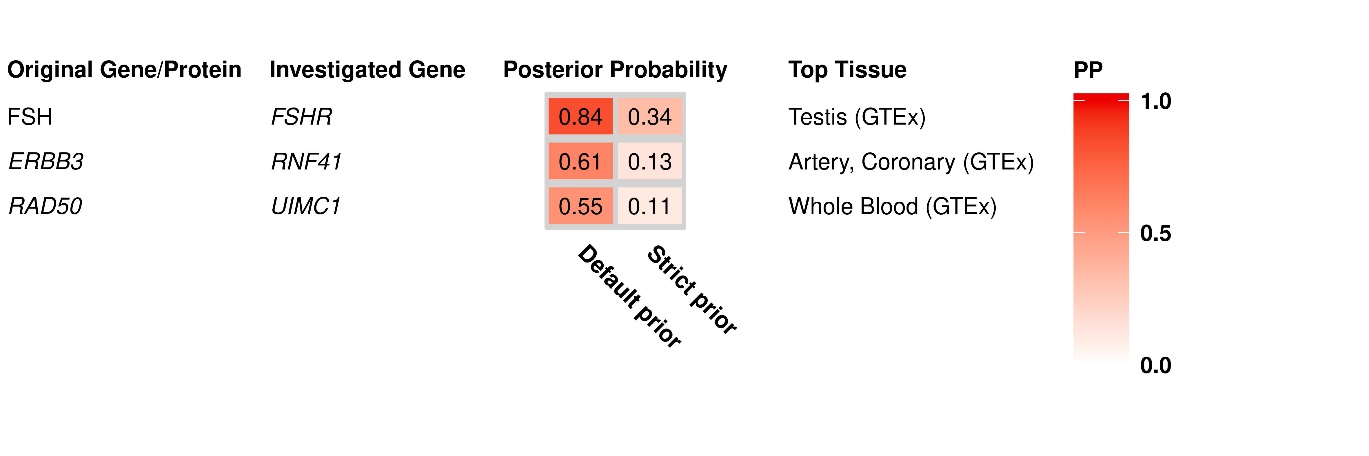


**Supplementary Figure 13. Posterior probabilities for genes with nominal evidence of colocalization in the interaction-Coloc analyses**

In the main approach, we used the main PCOS dataset and set the prior probability of colocalization to the default p12 = 1×10^-5^. Sensitivity analyses included a more stringent prior probability of p12 = 1×10^-6^. Note that *RNF41* – implicated in the same pathway as *ERBB3* – was also located in the rs2271194:A>T locus. PP, posterior probability.


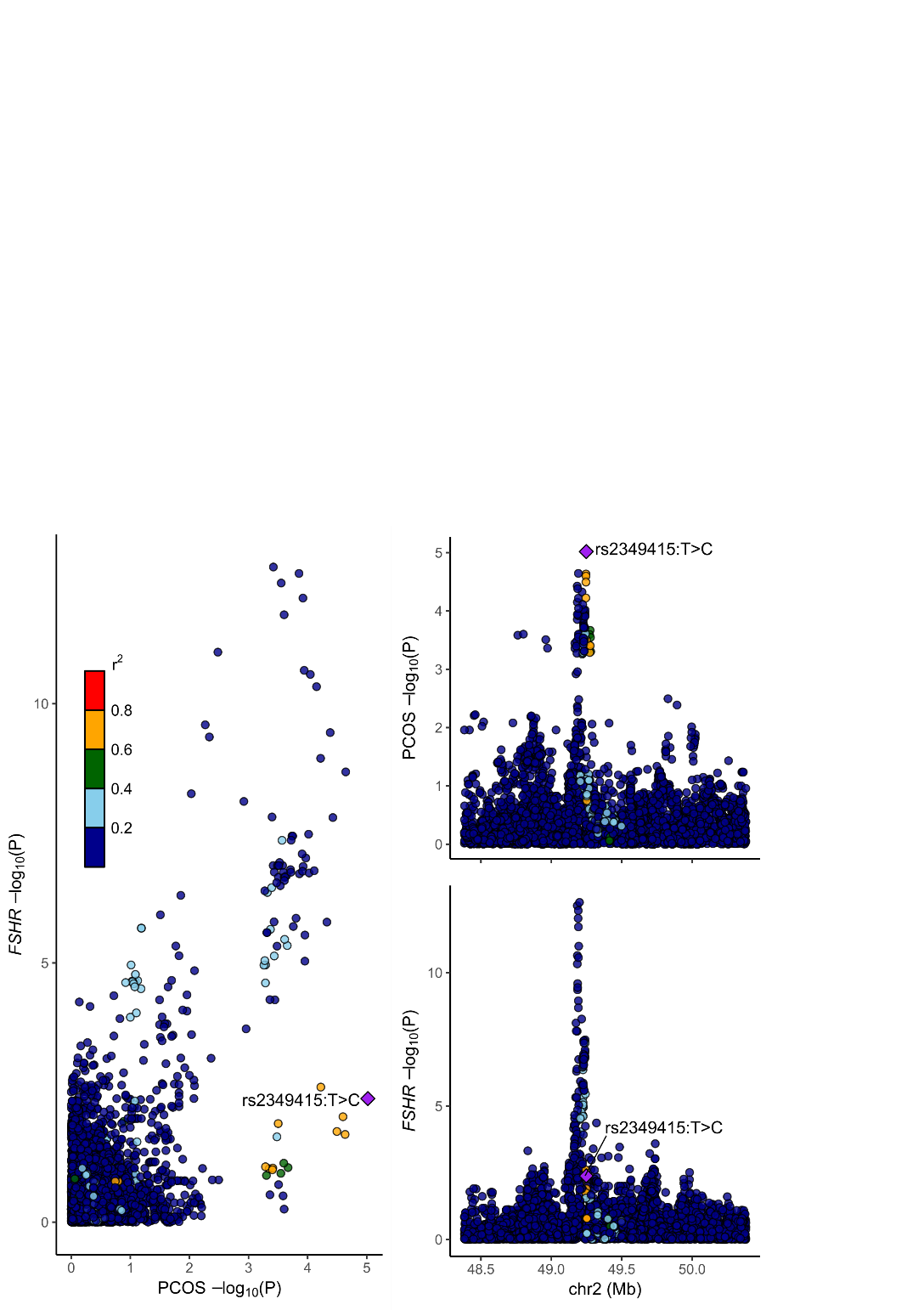


**Supplementary Figure 14. Associations between genetic variants and PCOS risk, using the main PCOS dataset and 2 Mb region sizes for *FSHR* expression levels in testis**

In each plot, each dot is a genetic variant. The SNP with the most significant P-value for PCOS is marked, with the other SNPs colour-coded according to linkage disequilibrium (r^2^) in Europeans with the lead variant. SNPs with missing linkage disequilibrium information are also coded dark blue. In the left panels, -log10 P-values for associations with PCOS risk are on the x-axes, and -log10 P-values for associations with the protein/transcript levels on the y-axes. On the right panels, genomic positions are on the x-axes, and the y-axes show -log10 P-values for PCOS on the upper panel and -log10 P-values with the protein/expression levels on the lower panel for the corresponding region. PCOS, polycystic ovary syndrome; SNP, single nucleotide polymorphism.


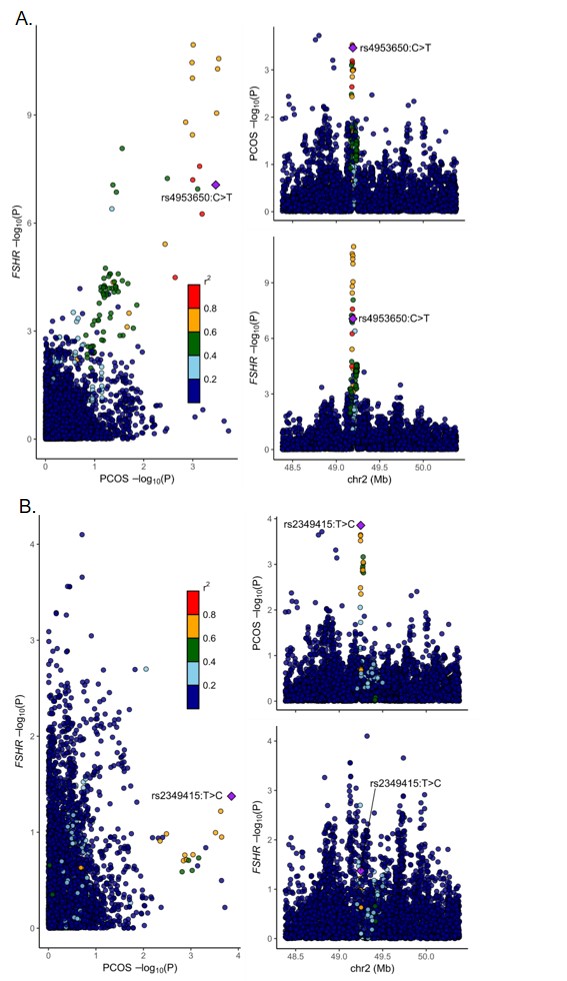


**Supplementary Figure 15A and 15B. Associations between genetic variants and PCOS risk, using the main PCOS dataset and 2 Mb region sizes for (A) *FSHR* expression levels in testis using estimates conditioned on rs2349415:T>C (B) *FSHR* expression levels in testis using estimates conditioned on rs4953650:C>T**

In each plot, each dot is a genetic variant. The SNP with the most significant P-value for PCOS in the unconditioned datasets is marked, with the other SNPs colour-coded according to linkage disequilibrium (r^2^) in Europeans with the lead variant. SNPs with missing linkage disequilibrium information are also coded dark blue. In the left panels, -log10 P-values for associations with PCOS risk are on the x-axes, and -log10 P-values for associations with the transcript levels on the y-axes. On the right panels, genomic positions are on the x-axes, and the y-axes show -log10 P-values for PCOS on the upper panel and -log10 P-values with the expression levels on the lower panel for the corresponding region. FSH, follicle stimulating hormone; PCOS, polycystic ovary syndrome; SNP, single nucleotide polymorphism.


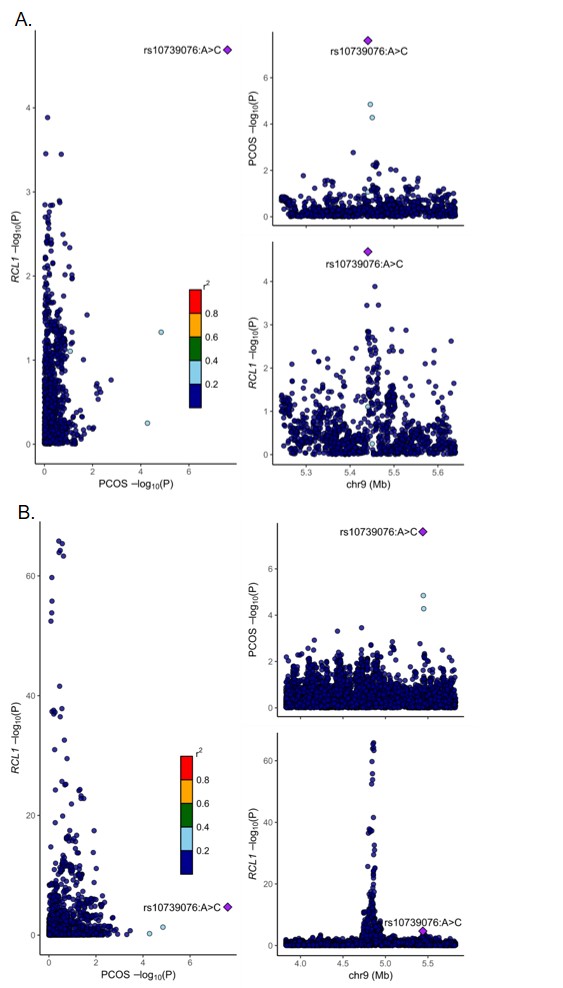


**Supplementary Figure 16A and 16B. Associations between genetic variants and PCOS risk, using the main PCOS dataset for *RCL1* expression levels in blood (eQTLgen) using (A) 400 kb region size (B) 2 Mb region size**

In each plot, each dot is a genetic variant. The SNP with the most significant P-value for PCOS is marked, with the other SNPs colour-coded according to linkage disequilibrium (r^2^) in Europeans with the lead variant. SNPs with missing linkage disequilibrium information are also coded dark blue. In the left panels, -log10 P-values for associations with PCOS risk are on the x-axes, and -log10 P-values for associations with the protein/transcript levels on the y-axes. On the right panels, genomic positions are on the x-axes, and the y-axes show -log10 P-values for PCOS on the upper panel and -log10 P-values with the protein/expression levels on the lower panel for the corresponding region. PCOS, polycystic ovary syndrome; SNP, single nucleotide polymorphism.

#

# References

1. Durinck S, Moreau Y, Kasprzyk A, Davis S, De Moor B, Brazma A, et al. BioMart and Bioconductor: a powerful link between biological databases and microarray data analysis. Vol. 21, Bioinformatics. 2005. p. 3439–40.

2. Durinck S, Spellman PT, Birney E, Huber W. Mapping identifiers for the integration of genomic datasets with the R/Bioconductor package biomaRt. Vol. 4, Nature Protocols. 2009. p. 1184–91.

3. Day F, Karaderi T, Jones MR, Meun C, He C, Drong A, et al. Large-scale genome-wide meta-analysis of polycystic ovary syndrome suggests shared genetic architecture for different diagnosis criteria. PLoS Genet. 2018 Dec 19;14(12):e1007813.

4. Sun BB, Maranville JC, Peters JE, Stacey D, Staley JR, Blackshaw J, et al. Genomic atlas of the human plasma proteome. Nature. 2018 Jun 6;558(7708):73–9.

5. GTEx Consortium. Genetic effects on gene expression across human tissues. Nature. 2017 Oct 12;550(7675):204–13.

6. Võsa U, Claringbould A, Westra H-J, Bonder MJ, Deelen P, Zeng B, et al. Unraveling the polygenic architecture of complex traits using blood eQTL metaanalysis. bioRxiv. 2018 Oct 19;447367.

7. Giambartolomei C, Vukcevic D, Schadt EE, Franke L, Hingorani AD, Wallace C, et al. Bayesian Test for Colocalisation between Pairs of Genetic Association Studies Using Summary Statistics. PLoS Genet. 2014 May 15;10(5):e1004383.

8. Myers TA, Chanock SJ, Machiela MJ. LDlinkR: An R Package for Rapidly Calculating Linkage Disequilibrium Statistics in Diverse Populations. Front Genet. 2020 Feb 28;11:157.

9. Auton A, Abecasis GR, Altshuler DM, Durbin RM, Bentley DR, Chakravarti A, et al. A global reference for human genetic variation. Nature. 2015;526(7571):68–74.

10. Foley CN, Staley JR, Breen PG, Sun BB, Kirk PDW, Burgess S, et al. A fast and efficient colocalization algorithm for identifying shared genetic risk factors across multiple traits. bioRxiv. 2019 Mar 28;592238.

11. Zhu Z, Zhang F, Hu H, Bakshi A, Robinson MR, Powell JE, et al. Integration of summary data from GWAS and eQTL studies predicts complex trait gene targets. Nat Genet. 2016 May;48(5):481–7.

12. Hormozdiari F, van de Bunt M, Segrè AV, Li X, Joo JWJ, Bilow M, et al. Colocalization of GWAS and eQTL Signals Detects Target Genes. Am J Hum Genet. 2016 Dec;99(6):1245–60.

13. Fabregat A, Jupe S, Matthews L, Sidiropoulos K, Gillespie M, Garapati P, et al. The Reactome Pathway Knowledgebase. Nucleic Acids Res. 2018 Jan 4;46(D1):D649–55.

14. CGA glycoprotein hormones, alpha polypeptide [Homo sapiens (human)] - Gene - NCBI [Internet]. [cited 2019 Jul 1]. Available from: https://www.ncbi.nlm.nih.gov/gene/1081

15. Sudlow C, Gallacher J, Allen N, Beral V, Burton P, Danesh J, et al. UK Biobank: An Open Access Resource for Identifying the Causes of a Wide Range of Complex Diseases of Middle and Old Age. PLoS Med. 2015 Mar 31;12(3):e1001779.

16. Bycroft C, Freeman C, Petkova D, Band G, Elliott LT, Sharp K, et al. The UK Biobank resource with deep phenotyping and genomic data. Nature. 2018 Oct;562(7726):203–9.

17. Purcell S, Neale B, Todd-Brown K, Thomas L, Ferreira MAR, Bender D, et al. PLINK: A Tool Set for Whole-Genome Association and Population-Based Linkage Analyses. Am J Hum Genet. 2007 Sep;81(3):559–75.

18. Machiela MJ, Chanock SJ. LDlink: a web-based application for exploring population-specific haplotype structure and linking correlated alleles of possible functional variants. Bioinformatics. 2015 Nov 1;31(21):3555–7.

19. Yang J, Lee SH, Goddard ME, Visscher PM. GCTA: A Tool for Genome-wide Complex Trait Analysis. Am J Hum Genet. 2011 Jan 7;88(1):76–82.

20. Yang J, Ferreira T, Morris AP, Medland SE, Madden PAF, Heath AC, et al. Conditional and joint multiple-SNP analysis of GWAS summary statistics identifies additional variants influencing complex traits. Nat Genet. 2012;44(4):369–75.

21. Berisa T, Pickrell JK. Approximately independent linkage disequilibrium blocks in human populations. Bioinformatics. 2016 Jan 15;32(2):283–5.

22. Sherry ST, Ward MH, Kholodov M, Baker J, Phan L, Smigielski EM, et al. dbSNP: the NCBI database of genetic variation. Nucleic Acids Res. 2001 Jan 1;29(1):308–11.

23. Carvalho-Silva D, Pierleoni A, Pignatelli M, Ong C, Fumis L, Karamanis N, et al. Open Targets Platform: new developments and updates two years on. Nucleic Acids Res. 2019 Jan 8;47(D1):D1056–65.

24. Ward LD, Kellis M. HaploReg: a resource for exploring chromatin states, conservation, and regulatory motif alterations within sets of genetically linked variants. Nucleic Acids Res. 2012 Jan 1;40(D1):D930–4.

25. R Core Team. R: A language and environment for statistical computing. R Foundation for Statistical Computing, Vienna, Austria [Internet]. 2017. Available from: https://www.r-project.org/

26. Free Software Foundation. bash 4.1.2(2) [Internet]. 2007. Available from: https://www.gnu.org/software/bash/

27. Free Software Foundation. GNU AWK 3.1.7 [Internet]. 1989. Available from: https://www.gnu.org/software/gawk/manual/gawk.html

28. Liu B, Gloudemans MJ, Rao AS, Ingelsson E, Montgomery SB. Abundant associations with gene expression complicate GWAS follow-up. Nat Genet. 2019 May 1;1.

29. Wickham H, Henry L. tidyr: Easily Tidy Data with “spread()” and “gather()” Functions [Internet]. 2018 [cited 2021 Jan 4]. Available from: https://CRAN.R-project.org/package=tidyr

30. Dowle M, Srinivasan A. data.table: Extension of `data.frame` [Internet]. 2020 [cited 2021 Jan 4]. Available from: https://CRAN.R-project.org/package=data.table

31. Wickham H. The Split-Apply-Combine Strategy for Data Analysis [Internet]. Vol. 40, Journal of Statistical Software. 2011. p. 1–29. Available from: http://www.jstatsoft.org/v40/i01/

32. Wickham H, Hester J, Chang W. devtools: Tools to Make Developing R Packages Easier [Internet]. 2019. Available from: https://CRAN.R-project.org/package=devtools

33. Wickham H. ggplot2: Elegant Graphics for Data Analysis. New York: Springer-Verlag; 2009.

34. UniProt Consortium. UniProt: a worldwide hub of protein knowledge. Nucleic Acids Res. 2019 Jan 8;47(D1):D506–15.

35. Grenon M, Gilbert C, Lowndes NF. Checkpoint activation in response to double-strand breaks requires the Mre11/Rad50/Xrs2 complex. Nat Cell Biol. 2001 Sep;3(9):844–7.

36. Theunissen J-WF, Kaplan MI, Hunt PA, Williams BR, Ferguson DO, Alt FW, et al. Checkpoint Failure and Chromosomal Instability without Lymphomagenesis in Mre11ATLD1/ATLD1 Mice. Mol Cell. 2003 Dec 1;12(6):1511–23.

37. Paull TT, Gellert M. The 3′ to 5′ Exonuclease Activity of Mre11 Facilitates Repair of DNA Double-Strand Breaks. Mol Cell. 1998 Jun 1;1(7):969–79.

38. Inagaki A, Roset R, Petrini JHJ. Functions of the MRE11 complex in the development and maintenance of oocytes. Chromosoma. 2016 Mar;125(1):151–62.

39. Trujillo KM, Yuan SS, Lee EY, Sung P. Nuclease activities in a complex of human recombination and DNA repair factors Rad50, Mre11, and p95. J Biol Chem. 1998 Aug 21;273(34):21447–50.

40. Roset R, Inagaki A, Hohl M, Brenet F, Lafrance-Vanasse J, Lange J, et al. The Rad50 hook domain regulates DNA damage signaling and tumorigenesis. Genes Dev. 2014 Mar 1;28(5):451–62.

41. Mailand N, Bekker-Jensen S, Faustrup H, Melander F, Bartek J, Lukas C, et al. RNF8 ubiquitylates histones at DNA double-strand breaks and promotes assembly of repair proteins. Cell. 2007 Nov 30;131(5):887–900.

42. Pan F, Yu H, Dang EV, Barbi J, Pan X, Grosso JF, et al. Eos mediates Foxp3-dependent gene silencing in CD4+ regulatory T cells. Science. 2009 Aug 28;325(5944):1142–6.

43. Gokhale AS, Gangaplara A, Lopez-Occasio M, Thornton AM, Shevach EM. Selective deletion of Eos (Ikzf4) in T-regulatory cells leads to loss of suppressive function and development of systemic autoimmunity. J Autoimmun. 2019 Jul 8;102300.

44. Kim J, Wu H-H, Lander AD, Lyons KM, Matzuk MM, Calof AL. GDF11 controls the timing of progenitor cell competence in developing retina. Science. 2005 Jun 24;308(5730):1927–30.

45. Liu J-P. The function of growth/differentiation factor 11 (Gdf11) in rostrocaudal patterning of the developing spinal cord. Development. 2006 Aug;133(15):2865–74.

46. Wu H-H, Ivkovic S, Murray RC, Jaramillo S, Lyons KM, Johnson JE, et al. Autoregulation of neurogenesis by GDF11. Neuron. 2003 Jan 23;37(2):197–207.

47. Luo H, Guo Y, Liu Y, Wang Y, Zheng R, Ban Y, et al. Growth differentiation factor 11 inhibits adipogenic differentiation by activating TGF-beta/Smad signalling pathway. Cell Prolif. 2019 Jul;52(4):e12631.

48. Harmon EB, Apelqvist AA, Smart NG, Gu X, Osborne DH, Kim SK. GDF11 modulates NGN3+ islet progenitor cell number and promotes beta-cell differentiation in pancreas development. Development. 2004 Dec;131(24):6163–74.

49. Li H, Li Y, Xiang L, Zhang J, Zhu B, Xiang L, et al. GDF11 Attenuates Development of Type 2 Diabetes via Improvement of Islet β-Cell Function and Survival. Diabetes. 2017 Jul;66(7):1914–27.

50. Padmanabhan V, Battaglia D, Brown MB, Karsch FJ, Lee JS, Pan W, et al. Neuroendocrine control of follicle-stimulating hormone (FSH) secretion: II. Is follistatin-induced suppression of FSH secretion mediated via changes in activin availability and does it involve changes in gonadotropin-releasing hormone secretion? Biol Reprod. 2002 May;66(5):1395–402.

51. Meriggiola MC, Dahl KD, Mather JP, Bremner WJ. Follistatin decreases activin-stimulated FSH secretion with no effect on GnRH-stimulated FSH secretion in prepubertal male monkeys. Endocrinology. 1994 Apr;134(4):1967–70.

52. Ueno N, Ling N, Ying SY, Esch F, Shimasaki S, Guillemin R. Isolation and partial characterization of follistatin: a single-chain Mr 35,000 monomeric protein that inhibits the release of follicle-stimulating hormone. Proc Natl Acad Sci U S A. 1987 Dec;84(23):8282–6.

53. Robertson DM, Klein R, de Vos FL, McLachlan RI, Wettenhall RE, Hearn MT, et al. The isolation of polypeptides with FSH suppressing activity from bovine follicular fluid which are structurally different to inhibin. Biochem Biophys Res Commun. 1987 Dec 16;149(2):744–9.

54. Gamer LW, Wolfman NM, Celeste AJ, Hattersley G, Hewick R, Rosen V. A novel BMP expressed in developing mouse limb, spinal cord, and tail bud is a potent mesoderm inducer in Xenopus embryos. Dev Biol. 1999 Apr 1;208(1):222–32.

55. El-Shafey M, Hegazy M, ElZahabi M, Farahat M. Clinical Signifiance of Follistatin in Obese and Non-obese Egyptian Polycystic Ovarian Patients. J Mol Pathophysiol. 2016;5(1):10.

56. Liu Y, Du S-Y, Ding M, Dou X, Zhang F-F, Wu Z-Y, et al. The BMP4-Smad signaling pathway regulates hyperandrogenism development in a female mouse model. J Biol Chem. 2017 Jul 14;292(28):11740–50.

57. Pan H, Ma P, Zhu W, Schultz RM. Age-associated increase in aneuploidy and changes in gene expression in mouse eggs. Dev Biol. 2008 Apr 15;316(2):397–407.

58. Biase FH. Oocyte Developmental Competence: Insights from Cross-Species Differential Gene Expression and Human Oocyte-Specific Functional Gene Networks. OMICS. 2017 Mar;21(3):156–68.

59. Martin RIR, Babaei MS, Choy M-K, Owens WA, Chico TJA, Keenan D, et al. Genetic variants associated with risk of atrial fibrillation regulate expression of PITX2, CAV1, MYOZ1, C9orf3 and FANCC. J Mol Cell Cardiol. 2015 Aug;85:207–14.

60. Castillo-Fernandez JE, Loke YJ, Bass-Stringer S, Gao F, Xia Y, Wu H, et al. DNA methylation changes at infertility genes in newborn twins conceived by in vitro fertilisation. Genome Med. 2017 Mar 24;9(1):28.

61. Xia L, Wang Y, Meng Q, Su X, Shen J, Wang J, et al. Integrated Bioinformatic Analysis of a Competing Endogenous RNA Network Reveals a Prognostic Signature in Endometrial Cancer. Front Oncol. 2019 May 29;9:448.

62. Chakraborty A, Wakamiya M, Venkova-Canova T, Pandita RK, Aguilera-Aguirre L, Sarker AH, et al. Neil2-null Mice Accumulate Oxidized DNA Bases in the Transcriptionally Active Sequences of the Genome and Are Susceptible to Innate Inflammation. J Biol Chem. 2015 Oct 9;290(41):24636–48.

63. Anurag M, Punturi N, Hoog J, Bainbridge MN, Ellis MJ, Haricharan S. Comprehensive Profiling of DNA Repair Defects in Breast Cancer Identifies a Novel Class of Endocrine Therapy Resistance Drivers. Clin Cancer Res. 2018 Oct 1;24(19):4887–99.

64. Rolseth V, Luna L, Olsen AK, Suganthan R, Scheffler K, Neurauter CG, et al. No cancer predisposition or increased spontaneous mutation frequencies in NEIL DNA glycosylases-deficient mice. Sci Rep. 2017 Jun 29;7(1):4384.

65. Martin JH, Aitken RJ, Bromfield EG, Nixon B. DNA damage and repair in the female germline: contributions to ART. Hum Reprod Update. 2019 Mar 1;25(2):180–201.

66. Jaroudi S, Kakourou G, Cawood S, Doshi A, Ranieri DM, Serhal P, et al. Expression profiling of DNA repair genes in human oocytes and blastocysts using microarrays. Hum Reprod. 2009 Oct;24(10):2649–55.

67. Schomacher L, Han D, Musheev MU, Arab K, Kienhöfer S, von Seggern A, et al. Neil DNA glycosylases promote substrate turnover by Tdg during DNA demethylation. Nat Struct Mol Biol. 2016 Feb;23(2):116–24.

68. Gu T-P, Guo F, Yang H, Wu H-P, Xu G-F, Liu W, et al. The role of Tet3 DNA dioxygenase in epigenetic reprogramming by oocytes. Nature. 2011 Sep 4;477(7366):606–10.

69. Horn DM, Mason SL, Karbstein K. Rcl1 protein, a novel nuclease for 18 S ribosomal RNA production. J Biol Chem. 2011 Sep 30;286(39):34082–7.

70. Paul P, van den Hoorn T, Jongsma MLM, Bakker MJ, Hengeveld R, Janssen L, et al. A Genome-wide multidimensional RNAi screen reveals pathways controlling MHC class II antigen presentation. Cell. 2011 Apr 15;145(2):268–83.

71. Huang X, Hao C, Shen X, Liu X, Shan Y, Zhang Y, et al. Differences in the transcriptional profiles of human cumulus cells isolated from MI and MII oocytes of patients with polycystic ovary syndrome. Reproduction. 2013 Jun;145(6):597–608.

72. Audano M, Pedretti S, Cermenati G, Brioschi E, Diaferia GR, Ghisletti S, et al. Zc3h10 is a novel mitochondrial regulator. EMBO Rep. 2018 Apr;19(4):e45531.

73. Kent WJ, Sugnet CW, Furey TS, Roskin KM, Pringle TH, Zahler AM, et al. The human genome browser at UCSC. Genome Res. 2002 Jun 1;12(6):996–1006.
